# Increasing incidence and changing trends in etiology & survival rates in HCC in a European cohort

**DOI:** 10.1016/j.jhepr.2026.101924

**Published:** 2026-06-23

**Authors:** Koen C. van Son, Kirsi M.A. van Eekhout, Judith de Vos-Geelen, Heinz-Josef Klümpen, Eric T.T.L. Tjwa, Joep de Bruijne, Nadia Haj Mohammad, Dave Sprengers, Minneke J. Coenraad, Frederike G.I. van Vilsteren, Otto M. van Delden, Joost P.H. Drenth, Maarten E. Tushuizen, Adriaan G. Holleboom, Lydia G. van der Geest, R. Bart Takkenberg, for the Dutch Hepatocellular & Cholangio Carcinoma Group (DHCG)

**Affiliations:** 1 Department of Vascular Medicine, Amsterdam UMC, University of Amsterdam, Meibergdreef 9, 1105 AZ, Amsterdam, The Netherlands; 2 Department of Gastroenterology and Hepatology, Amsterdam UMC, University of Amsterdam, De Boelelaan 1117, 1081 HV Amsterdam, The Netherlands; 3 Amsterdam Gastroenterology Endocrinology Metabolism (AGEM) Institute, Amsterdam UMC, University of Amsterdam, Meibergdreef 9, 1105 AZ, Amsterdam, The Netherlands; 4 Div. Medical Oncology, Department of Internal Medicine, GROW – Research Institute for Oncology & Reproduction, Maastricht University Medical Center, P. Debyelaan 25, 6229 HX, Maastricht, The Netherlands; 5 Department of Medical Oncology, UMC Groningen, Hanzeplein 1, 9713 GZ Groningen, The Netherlands; 6 Department of Gastroenterology and Hepatology, Radboud University Medical Center, Geert Grooteplein Zuid 10, 6525 GA, Nijmegen, The Netherlands; 7 Department of Gastroenterology and Hepatology, Utrecht University Medical Center, Utrecht, Heidelberglaan 100, 3584 CV, Utrecht, The Netherlands; 8 Department of Medical Oncology, Utrecht University Medical Center, Utrecht University, Utrecht, Heidelberglaan 100, 3584 CV, Utrecht, The Netherlands; 9 Department of Gastroenterology and Hepatology, Erasmus University Medical Center, Dr. Molewaterplein 40, 3015 GD, Rotterdam, The Netherlands; 10 Department of Gastroenterology and Hepatology, Leiden University Medical Center, Albinusdreef 2, 2333 ZA, Leiden, The Netherlands; 11 Department of Gastroenterology, Division of Hepatology, University Medical Center Groningen, Hanzeplein 1, 9713 GZ, Groningen, The Netherlands; 12 Department of Radiology, Amsterdam UMC, University of Amsterdam, De Boelelaan 1117, 1081 HV Amsterdam, The Netherlands; 13 Department of Research and Development, Netherlands Comprehensive Cancer Organisation (IKNL), Rijnkade 5, 3511 LC, Utrecht, the Netherlands

**Keywords:** Hepatocellular carcinoma, temporal trends, epidemiology, nationwide, registry

## Abstract

**Background & Aims:**

Hepatocellular carcinoma (HCC) represents a major global health burden. While incidence rates are declining in some regions, they are rising in the Netherlands, traditionally a low-endemic country for HCC. Despite these trends, comprehensive data on longitudinal changes in etiology, tumor characteristics, and overall survival remain scarce.

**Methods:**

Consecutive patients registered in the Netherlands Cancer Registry with newly diagnosed HCC were included (2014-2022). Average annual percentage change (AAPC) was calculated to assess trends in age-adjusted incidence rates. Cox regression analyses were performed to determine risk factors for overall mortality.

**Results:**

Between January 2014 and December 2022, 6,526 patients were diagnosed with HCC in the Netherlands. The age-adjusted incidence rate of HCC rose from 3.5 to 4.1 per 100,000 person-years, reflecting an AAPC of 2.3% (95% CI 0.9-3.7). While alcohol-related liver disease (ALD) was the most common etiology of HCC, metabolic dysfunction-associated steatotic liver disease (MASLD)-related HCC had the highest AAPC at 8.3% (95% CI 2.9-13.7). 1-, 3- and 5-year survival rates were 46.5%, 26.1% and 18.8%, respectively. ALD-related HCC was associated with worse overall survival probability than MASLD-related HCC (hazard ratio [HR] 1.21; 95% CI 1.08-1.35). Curative-intent treatment allocation had the most profound effect on overall survival probability (HR 0.17; 95% CI 0.15-0.19), followed by other tumor-directed treatment (HR 0.37; 95% CI 0.34-0.40), and the presence of distant metastases (HR 1.99; 95% CI 1.83-2.15).

**Conclusion:**

This comprehensive nationwide cohort study demonstrates a sustained increase in HCC incidence rates in the Netherlands, with a significant rise in MASLD-related HCC. Overall survival remains poor. These findings underscore the need for increased clinical awareness and further investigation into surveillance strategies.

**Impact and implications:**

Hepatocellular carcinoma (HCC) represents a major global health problem. However, comprehensive data on longitudinal changes from nationwide cohorts remain limited. This study demonstrates that the incidence rate of HCC is increasing in the Netherlands. Furthermore, it identifies treatment allocation, the presence of distant metastases, Child-Pugh C, and multifocal disease as key predictors of overall mortality. Overall survival remains poor, particularly among patients with no registered risk factor for chronic liver disease or those with unknown cirrhotic status. These findings underscore the need for increased clinical awareness and early diagnostic evaluation of underlying liver disease to facilitate timely detection, appropriate management, and improved clinical outcomes.

## Highlights

• Age-adjusted HCC incidence in the Netherlands rose by 2.3% annually (2014-2022).
• MASLD-related HCC increased almost 10% annually over the same time period.
• While overall survival was poor, MASLD-related HCC was associated with better survival probability than ALD-related HCC.
• Treatment was the strongest predictor of mortality, followed by distant metastases.

## Graphical abstract

**Figure:**
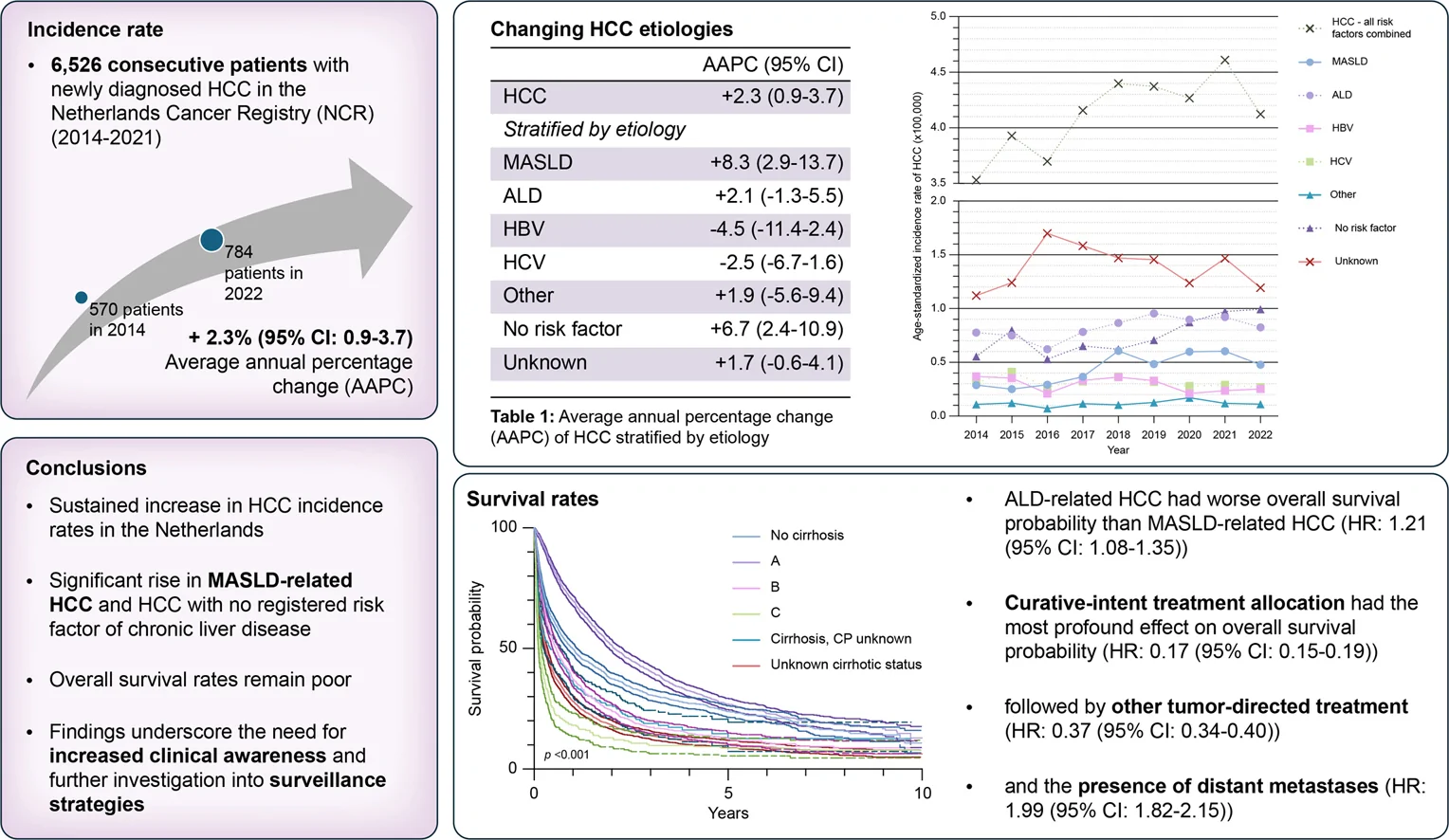

## Introduction

Primary liver cancer is the eighth most prevalent malignancy globally and the third leading cause of cancer-related mortality.^1^ Hepatocellular carcinoma (HCC) accounts for approximately 90% of all primary liver cancers and represents a major global health burden.^2^ While HCC incidence rates are declining in certain regions, they are increasing in traditionally low endemic countries such as the Netherlands.^3^^,^^4^ Despite these trends, comprehensive data on longitudinal changes in etiology, tumor characteristics, and overall survival among patients with HCC remain scarce.

The primary risk factor for HCC is chronic liver disease, most notably hepatitis B virus (HBV) and hepatitis C virus (HCV) infections, alcohol-related liver disease (ALD), and metabolic dysfunction-associated steatotic liver disease (MASLD).^5^ Globally, HBV and HCV remain the most common etiologies for HCC. However, the widespread adoption of HBV vaccination and improved antiviral therapies for HCV have led to a decline in virus-associated HCC incidence.^6^ Conversely, the incidence and prevalence of MASLD are on the rise, currently affecting approximately 30% of adults worldwide,^7^ driven by the increasing prevalence of type 2 diabetes mellitus and obesity.^8^ As a consequence, the incidence of MASLD-related HCC is growing, with projections suggesting increases ranging from 44% in Japan to 122% in the United States by 2030.^9^

Despite these shifts, the literature on overall survival rates of patients with MASLD-related HCC remains conflicting. Some studies report poorer overall survival for patients with MASLD-related HCC compared to those with other etiologies,[10], [11], [12] while other studies find no significant differences.^13^^,^^14^ Additionally, a significant clinical challenge with MASLD-related HCC is its development in the absence of cirrhosis in approximately 40% of cases. Since surveillance programs typically rely on the presence of cirrhosis, early detection of HCC in this population is often hampered.^15^

Most existing data are derived from specialized centers or specific cancer registries, which may not fully reflect routine clinical practice. Therefore, this study aimed to investigate the incidence, etiology, tumor characteristics, and overall survival of patients with HCC using data from the nationwide Netherlands Cancer Registry (NCR) between 2014 and 2022.

## Patients and methods

### Study population and data collection

All patients registered with HCC in the NCR from January 2014 to December 2022 were included. The NCR is a nationwide population-based cancer registry that covers the entire Dutch population of approximately 18 million people. Cancer diagnoses were notified to the NCR from the Dutch Nationwide Pathology Archives (PALGA) and the Dutch National Hospital Care Registry (*i.e.* hospital discharges and outpatient visits). All cases were confirmed through verification of patient records in all Dutch hospitals by trained registration clerks approximately 9 months after diagnosis. Vital status was updated annually by linkage with the nationwide municipal administrative database. HCC was defined according to the ICD-O-3 (International Classification of Diseases for Oncology Third Edition) topography C22.0 combined with morphology codes (Table S1).^16^ Overall survival was defined as the time from diagnosis to death (any cause) or the end of follow-up (emigration or 1 February 2024). When multiple risk factors for HCC were present, a hierarchical order was applied to determine which etiology was recorded (Table S1). The presence of cirrhosis was determined based on pathological reports or, if pathology was unavailable, on radiological assessment. If reports did not contain clear information about its presence or absence, registration clerks recorded ‘unknown’. Curative-intent treatment was defined as resection, primary tumor destruction using radiofrequency ablation or microwave ablation, or liver transplantation. Other tumor-directed treatment was defined as any tumor-directed therapy that did not meet the aforementioned curative criteria (Table S1). All data were pseudoanonymized. The study proposal was evaluated by the Dutch Hepatocellular & Cholangiocarcinoma Group and the Privacy Review Board of the NCR (K22.269). The Institutional Medical Ethics Committee of the Amsterdam University Medical Center waived the need for ethical approval and individual informed consent. The reporting of this study followed the STROBE (Strengthening the Reporting of Observational Studies in Epidemiology) guidelines.^17^

### Statistical analysis

Statistical analyses were performed using R (version 4.2.1; R Foundation for Statistical Computing, Vienna, Austria). *p* values <0.05 were considered statistically significant. Patient characteristics were expressed as frequencies with percentages, means (±SD) or medians (IQR), depending on the data distribution. Data were stratified by year of diagnosis, etiology (MASLD, ALD, HBV, HCV, other (auto-immune hepatitis, primary biliary cirrhosis, hemochromatosis, Wilson’s disease, porphyria), no risk factor for chronic liver disease and unknown), and presence of cirrhosis (yes, no, unknown) (Table S1). Age-standardized incidence rates per 100,000 person-years were calculated using the revised European Standard Population.^18^ Trends in these rates were analyzed using log-linear Joinpoint regression analysis and summarized using the average annual percentage change (AAPC) with a 95% CI. Boxplots and Kruskal-Wallis tests were utilized to compare median tumor diameters stratified by risk factor and cirrhosis status (yes, no, unknown).

Kaplan-Meier survival curves, 1-, 3-, and 5-year survival rates, and log-rank tests were used to compare overall survival probability stratified by sex, etiology, and Child-Pugh (CP) score. Cox regression analyses were used to evaluate possible predictors of better survival, *e.g*. sex, age at diagnosis, etiology, CP score, tumor diameter, number of nodules, the presence of distant metastases, and treatment allocation Table 1.

**Table 1:** Patient characteristics of NCR HCC cohort (N = 6,526).

	Total patient cohort (N = 6,526)
Year of diagnosis, n (%)	
2014	570 (8.7)
2015	646 (9.9)
2016	624 (9.6)
2017	714 (10.9)
2018	767 (11.8)
2019	786 (12.0)
2020	777 (11.9)
2021	858 (13.1)
2022	784 (12.0)
Sex, n men (%)	4,901 (75.1)
T2DM, n (%)	
Yes	1,794 (27.5)
No	2,380 (36.5)
Unknown	2,352 (36.0)
CVD, n (%)	
Yes	1,053 (16.1)
No	3,121 (47.8)
Unknown	2,352 (36.0)
Age at diagnosis, years (mean (SD))	69.8 (10.6)
Etiology, n (%)	
MASLD	711 (10.9)
ALD	1,322 (20.3)
HBV	466 (7.1)
HCV	508 (7.8)
Other	186 (2.9)
No risk factor	1,175 (18.0)
Unknown	2,158 (33.1)
Cirrhosis, n (%)	
Yes	3,223 (49.4)
No	1,676 (25.7)
Unknown	1,627 (24.9)
CP score, n (%)	
A	1,850 (28.3)
B	864 (13.2)
C	250 (3.8)
Cirrhosis, CP score unknown	259 (4.0)
No cirrhosis	1,676 (25.7)
Unknown cirrhotic status	1,627 (24.9)
Tumor diameter, mm (median (IQR))	50 (28, 91)
Number of nodules, n (%)	
1	2,858 (43.8)
2	854 (13.1)
3	390 (6.0)
Multifocal/diffuse	1,938 (29.7)
Unknown	486 (7.4)
Distant metastasis, n (%)	
Yes	1,263 (19.4)
No	5,097 (78.1)
Unknown	166 (2.5)
Treatment, n (%)	
Curative-intent	1,762 (27.0)
Other tumor-directed	1,719 (26.3)
No tumor-directed	3,045 (46.7)

ALD, alcohol-related liver disease; CP, Child–Pugh; CVD, cardiovascular disease; HBV, hepatitis B virus; HCV, hepatitis C virus; MASLD, metabolic dysfunction-associated steatotic liver disease; T2DM, type 2 diabetes mellitus.

## Results

**Fig. 1:**
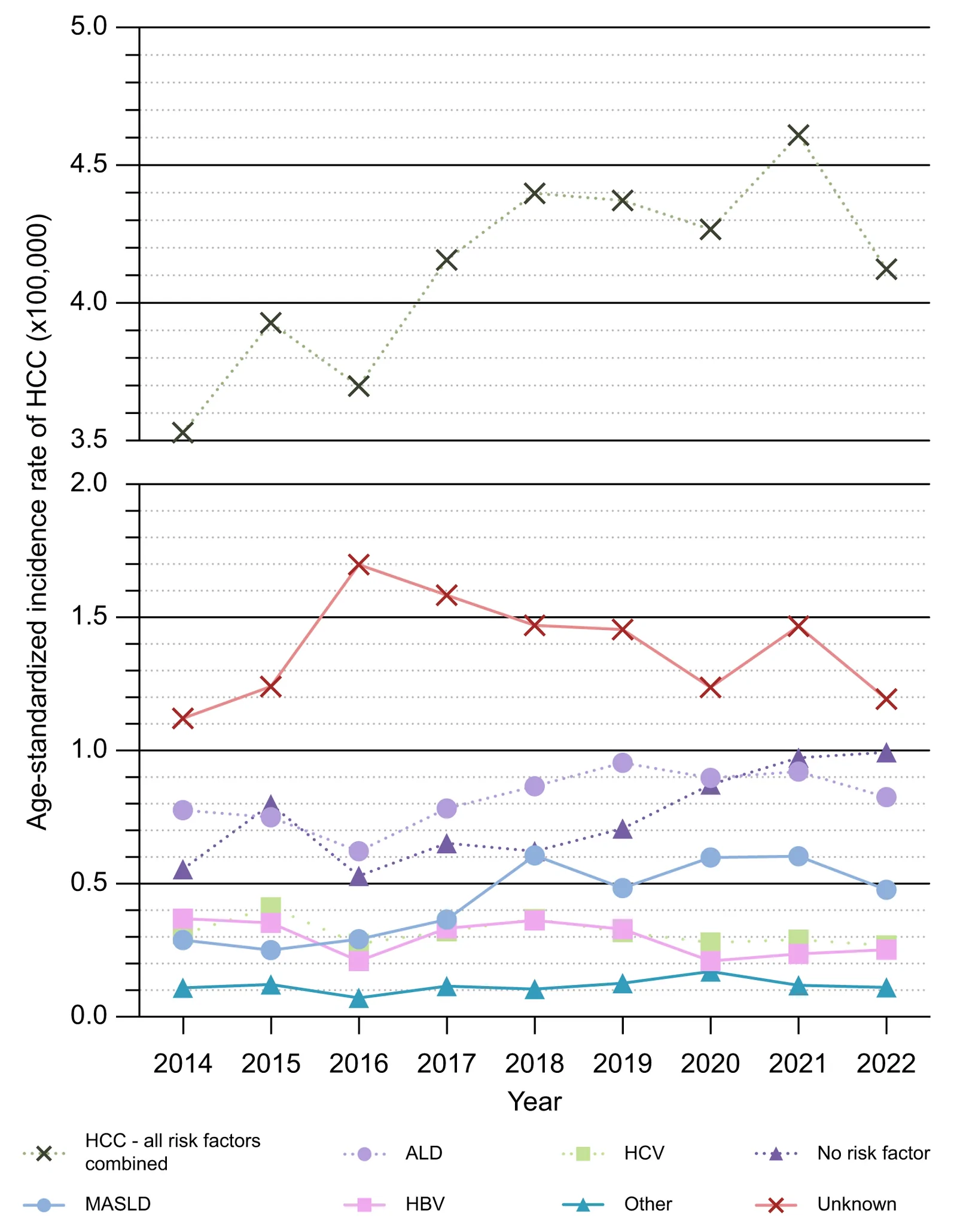
**Age-standardized incidence rate of HCC, and HCC stratified by etiology (N = 6,526).** ALD, alcohol-related liver disease; HBV, hepatitis B virus; HCC, hepatocellular carcinoma; HCV, hepatitis C virus; MASLD, metabolic dysfunction-associated steatotic liver disease.

Between January 2014 and December 2022, 6,526 patients were diagnosed with HCC in the Netherlands. The annual number of HCC diagnoses increased from 570 in 2014 to 784 in 2022, with a peak of 858 diagnoses in 2021. The age-adjusted incidence rate rose from 3.5 to 4.1 per 100,000 person-years, corresponding to an AAPC of 2.3% (95% CI 0.9-3.7) (Fig. 1; Table 2). Mean age at diagnosis was 69.8 s (±10.6) years, and 75.1% of patients were men (Table 1).

**Table 2:** Average annual percentage change of HCC in the Netherlands between 2014 and 2022 stratified by etiology (N = 6,526).

	Age-adjusted incidence rate (per 100,000 person-years)	Age-adjusted incidence rate (per 100,000 person-years)	AAPC (95% CI)
	2014	2022	
HCC	3.53	4.12	2.3 (0.9-3.7)

**Stratified by etiology**			
MASLD	0.29	0.48	8.3 (2.9-13.7)
ALD	0.78	0.83	2.1 (-1.3 to 5.5)
HBV	0.37	0.25	-4.5 (-11.4 to 2.4)
HCV	0.31	0.27	-2.5 (-6.7 to 1.6)
Other	0.11	0.11	1.9 (-5.6 to 9.4)
No risk factor	0.55	0.99	6.7 (2.4-10.9)
Unknown	1.12	1.19	1.7 (-0.6 to 4.1)

AAPC, average annual percentage change; ALD, alcohol-related liver disease; HBV, hepatitis B virus; HCC, hepatocellular carcinoma; HCV, hepatitis C virus; MASLD, metabolic dysfunction-associated steatotic liver disease.

**Fig. 2:**
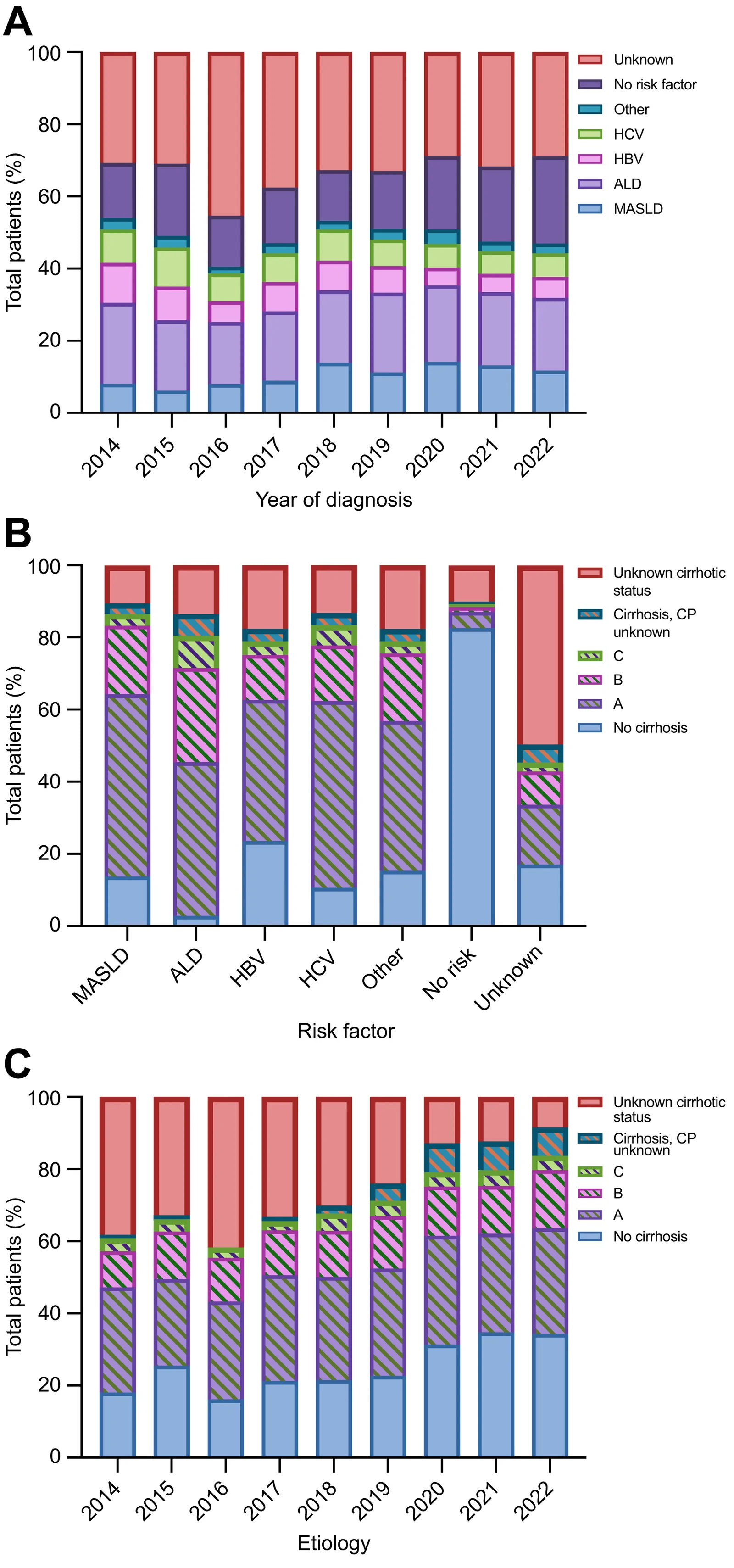
**Temporal trends in liver disease etiology and liver function at** HCC **diagnosis.** Etiology by year of diagnosis (N = 6,526) (A), CP score stratified by etiology (N = 6,526) (B), and CP score per year of inclusion (N = 6,526) (C). Diagonal lines indicate the presence of cirrhosis. No risk = no risk factor for chronic liver disease. ALD, alcohol-related liver disease; CP, Child-Pugh; HBV, hepatitis B virus; HCC, hepatocellular carcinoma; HCV, hepatitis C virus; MASLD, metabolic dysfunction-associated steatotic liver disease.

**Fig. 3:**
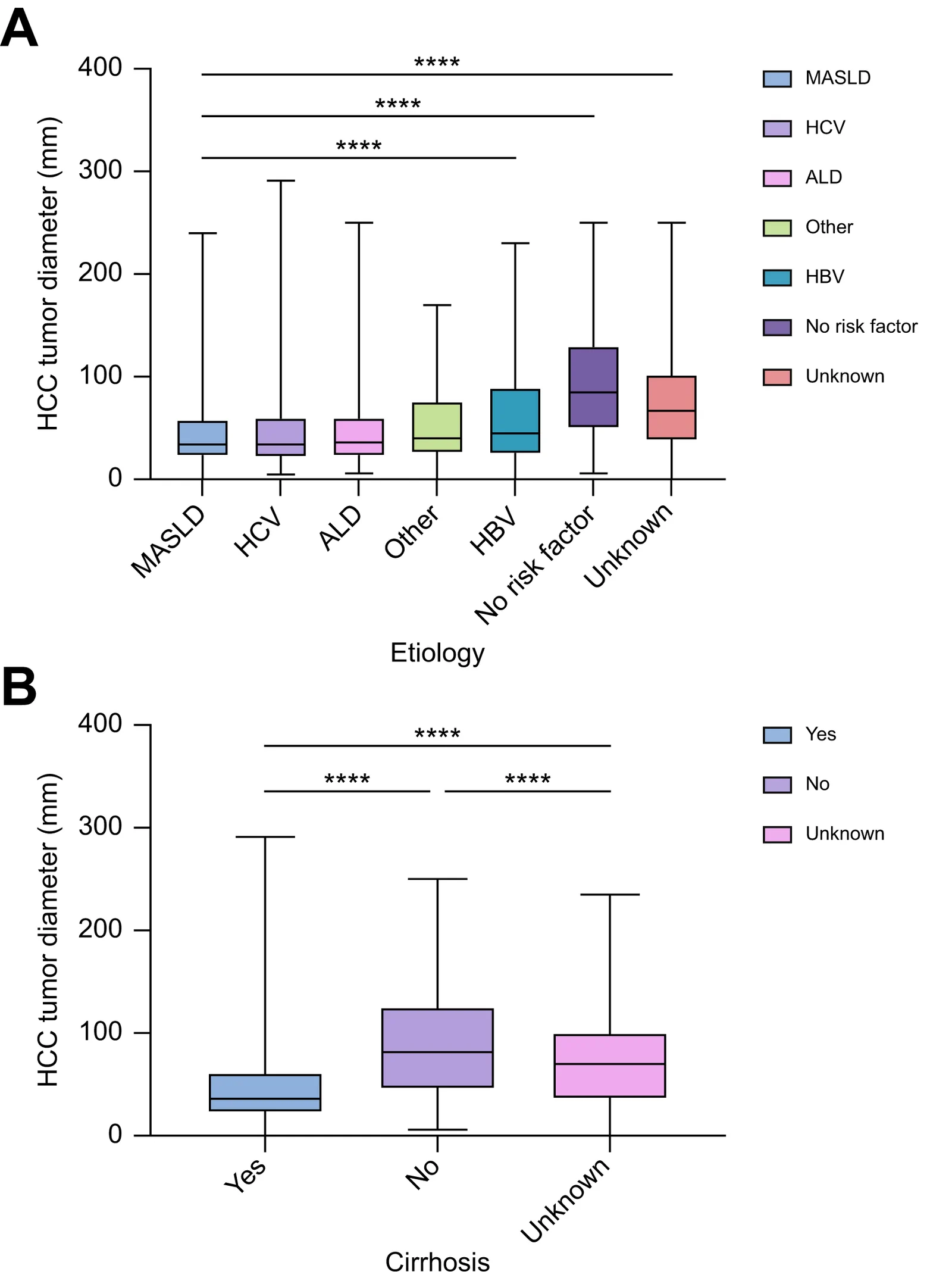
**Mean tumor diameter stratified by etiology and cirrhosicstatus.** Mean tumor diameter (bars represent SD; bold lines inside the box plot represent medians) stratified by etiology (n = 5,490) (A), and by cirrhosis status (n = 5,490) (B). Levels of significance: ∗∗∗∗*p* <0.001, ∗∗∗*p* <0.005, ∗∗*p* <0.01, ∗*p* <0.05 (Kruskal-Wallis test). ALD, alcohol-related liver disease; HBV, hepatitis B virus; HCC, hepatocellular carcinoma; HCV, hepatitis C virus; MASLD, metabolic dysfunction-associated steatotic liver disease.

ALD was the most common etiology of HCC (20.3%), followed by MASLD (10.9%), HCV (7.8%), HBV (7.1%), and other causes (2.9%). No underlying risk factor for chronic liver disease was found in 18.0% of patients, while etiology was unknown in 33.1% (Fig. 2A; Table 1). Fig. 1 shows age-adjusted HCC incidence rates stratified by etiology. MASLD-related HCC had the highest AAPC (8.3%; 95% CI 2.9-13.7) followed by HCC among patients registered as having no chronic liver disease (6.7%; 95% CI 2.4-10.9) (Tables 2 and S2). Other etiologies showed no significant trend over time (Fig. 1; Table 2). Patients without a registered risk factor for chronic liver disease and patients with unknown etiology presented with larger median tumor diameters compared to patients with any known etiology (Fig. 3A; Table S3). Patients with ALD-related HCC more often had multifocal (31.6% *vs.* 22.4%, *p* <0.001) and metastatic disease (12.9% *vs.* 8.9%, *p =* 0.007) than those with MASLD-related HCC (Table S3). In total, 27.0% of all patients received treatment with curative intent, 26.3% received other tumor-directed treatment, and 46.7% received no tumor-directed treatment (Table 1). Patients with MASLD-related HCC more often received curative-intent and other tumor-directed treatment than those with ALD-related HCC (39.1% *vs.* 29.0%, *p* <0.001 and 34.3% *vs.* 29.0%, *p* <0.001, respectively) (Table S3).

Cirrhosis was present in 3,223 (49.4%) patients, of whom 1,850 (57.4%) had CP-A, 864 (26.8%) had CP-B, and 250 (7.7%) had CP-C. Cirrhosis was absent in 1,676 (25.7%) patients, while cirrhotic status was unknown in 1,627 (24.9%) patients (Table 1). Among the 711 patients with MASLD-related HCC, cirrhosis was present in 538 (75.7%) patients and absent in 98 (13.8%) patients (Table S3; Fig. 2B). Patients with MASLD- and HCV-related HCC were more likely to have CP-A compared to those with ALD-related HCC. CP-C was more common among patients with ALD-related HCC than those with other etiologies (Fig. 2B; Table S3). The proportion of cases with unknown cirrhotic status decreased from over 30% to approximately 10% during the final 3 years of inclusion (Fig. 2C). Median tumor diameter was larger in patients without cirrhosis compared to those with cirrhosis (80 mm [IQR 46-122] *vss.* 36 mm [IQR 23-62], *p* <0.001) (Fig. 3B*;* Table S4). Patients without cirrhosis more frequently had distant metastases (22.6% *vs.* 13.4%, *p* <0.001) compared to those with cirrhosis. However, the proportion of patients receiving curative-intent treatment was similar between the two groups (30.1% *vs.* 31.1%, *p =* 0.700) (Table S4). Patients with cirrhosis more often received other tumor-directed treatment compared to those without cirrhosis (32.0% *vs.* 28.3%, *p =* 0.005).

**Fig. 4:**
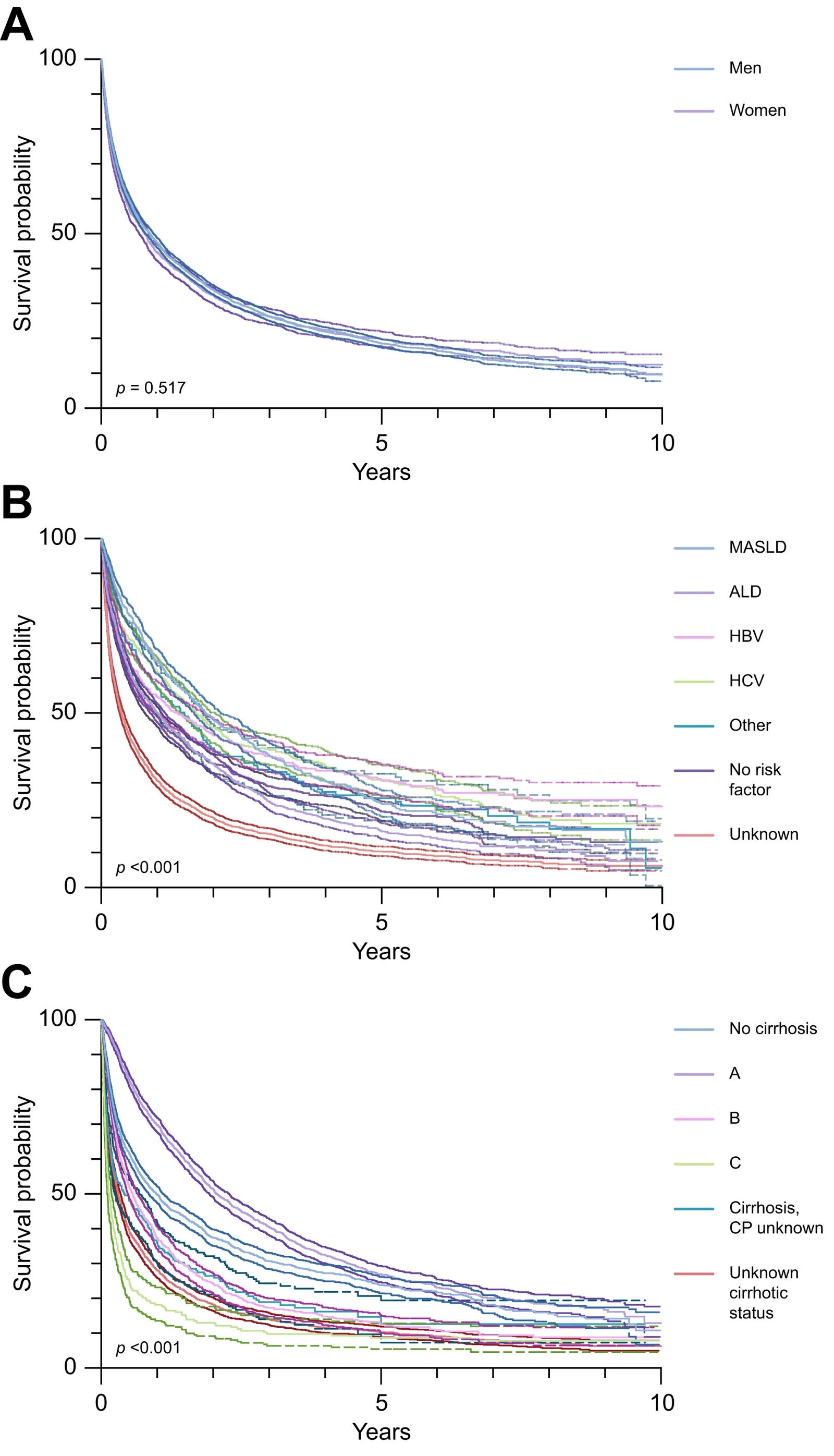
Kaplan-Meier survival curve stratified by sex, etiology and CP score. Kaplan-Meier survival curve (bold lines represent survival probability; dashed lines represent 95% confidence intervals) stratified by sex (N = 6,526) (log-rank *p =* 0.493) (A), stratified by etiology (N = 6,526) (log-rank *p* <0.001) (B), and stratified by Child-Pugh score (N = 6,526) (log-rank *p* <0.001) (C). ALD, alcohol-related liver disease; CP, Child-Pugh; HBV, hepatitis B virus; HCV, hepatitis C virus; MASLD, metabolic dysfunction-associated steatotic liver disease.

The 1-, 3-, and 5-year overall survival rates in the entire patient cohort were 46.5, 26.1 and 18.8%, respectively (Table S5). No difference in overall survival was observed between sexes (log-rank *p =* 0.530), nor was survival associated with year of diagnosis (log-rank *p =* 0.287) (Fig. 4A). One-, 3-, and 5-year overall survival rates were higher among patients with MASLD-related HCC (65.3%, 37.9% and 24.3%) compared to patients with ALD-related HCC (50.0%, 24.7% and 16.1%, log-rank *p* <0.001), patients registered as having no chronic liver disease (48.8%, 28.9% and 21.9%, log-rank *p* <0.001) and patients with unknown etiology (30.7%, 15.5% and 10.6%, log-rank *p* <0.001) (Fig. 4B; Table S5). When stratified by CP score, overall survival was poorest among those with CP-C (Fig. 4C; Table S5).

Age, sex, etiology, CP score, tumor diameter, number of nodules, the presence of distant metastases, and treatment allocation were included in a multivariable Cox regression analysis (Table 3). Allocation of curative-intent treatment was associated with the strongest reduction in mortality risk (hazard ratio [HR] 0.17; 95% CI 0.15-0.19), followed by other tumor-directed treatment (HR 0.37; 95% CI 0.34-0.40). In contrast, the presence of distant metastases (HR 1.99; 95% CI 1.83-2.15) and CP-C (HR 1.90; 95% CI 1.57-2.29) were strongly associated with increased mortality risk. ALD-related HCC was associated with an increased risk of mortality compared to MASLD-related HCC (HR 1.21; 95% CI 1.08-1.35). Similarly, unknown etiology was associated with an increased risk of mortality compared to MASLD-related HCC (HR 1.17; 95% CI 1.05-1.31).

**Table 3:** Univariable and multivariable Cox regression analysis of patient and tumor characteristics and mortality (N = 6,526).

Variables	Univariable Cox regression	Multivariable Cox regression	HR (95% CI)
	Patients (N = 6,526)	HR (95% CI)	
Sex, n (%)			
Men	4,901 (75.1)	Ref.	Ref.
Women	1,625 (24.9)	1.021 (0.958-1.087)	1.000 (0.931-1.075)
Age at diagnosis, years (mean (SD))	69.8 (10.6)	1.029 (1.027-1.032)	1.012 (1.009-1.016)
Etiology, n (%)			
MASLD	711 (10.9)	Ref.	Ref.
ALD	1,322 (20.3)	1.424 (1.280-1.584)	1.208 (1.078-1.352)
HBV	466 (7.1)	1.038 (0.902-1.195)	1.013 (0.869-1.179)
HCV	508 (7.8)	1.007 (0.879-1.154)	0.926 (0.800-1.073)
Other	186 (2.9)	1.138 (0.943-1.374)	0.961 (0.786-1.174)
No risk factor	1,175 (18.0)	1.312 (1.175-1.465)	0.915 (0.800-1.046)
Unknown	2,158 (33.1)	2.109 (1.911-2.329)	1.172 (1.047-1.312)
CP score, n (%)			
No cirrhosis	1,676 (25.7)	Ref.	Ref.
A	1,850 (28.3)	0.732 (0.677-0.791)	0.991 (0.894-1.100)
B	864 (13.2)	1.395 (1.273-1.528)	1.432 (1.269-1.616)
C	250 (3.8)	2.404 (2.086-2.770)	1.895 (1.571-2.286)
Cirrhosis, CP score unknown	259 (4.0)	1.525 (1.316-1.766)	1.336 (1.120-1.595)
Unknown cirrhotic status	1,627 (24.9)	1.777 (1.648-1.917)	1.100 (0.992-1.220)
Tumor diameter, mm (median (IQR))	50 (28, 91)	1.009 (1.008-1.009)	1.005 (1.005-1.006)
Number of nodules, n (%)			
1	2,858 (43.8)	Ref.	Ref.
2	854 (13.1)	1.225 (1.120-1.339)	1.170 (1.067-1.283)
3	390 (6.0)	1.373 (1.219-1.547)	1.146 (1.012-1.297)
Multifocal/ diffuse	1,938 (29.7)	2.930 (2.744-3.129)	1.471 (1.362-1.588)
Unknown	486 (7.4)	2.317 (2.008-2.572)	1.235 (0.969-1.574)
Distant metastasis, n (%)			
No	5,097 (78.1)	Ref.	Ref.
Yes	1,263 (19.4)	3.680 (3.438-3.940)	1.986 (1.831-2.154)
Unknown	166 (2.5)	4.247 (3.630-4.970)	1.511 (1.077-2.120)
Treatment, n (%)			
No tumor-directed	3,045 (46.7)	Ref.	
Curative-intent	1,762 (27.0)	0.093 (0.086-0.101)	0.169 (0.153-0.187)
Other tumor-directed	1,719 (26.3)	0.287 (0.268-0.307)	0.372 (0.344-0.403)

ALD, alcohol-related liver disease; CP, Child-Pugh; HBV, hepatitis B virus; HCC, hepatocellular carcinoma; HCV, hepatitis C virus; MASLD, metabolic dysfunction-associated steatotic liver disease.

## Discussion

Here we present data on incidence, etiology, tumor characteristics, and overall survival of patients with HCC in the Netherlands between 2014 and 2022. The age-adjusted incidence rate rose from 3.5 to 4.1 per 100,000 person-years, corresponding to an AAPC of 2.3%. Although ALD was the most common etiology, MASLD-related HCC showed the strongest increase over time. Incidence rates for other etiologies remained stable throughout the study period.

Our findings build on the timeline of earlier Dutch HCC cohort studies. Between 1989 and 2009, the AAPC in HCC incidence was 0.6%,^19^ whereas data from 2009-2016 showed an increase in the age-adjusted incidence rate from 2.9 to 3.7 per 100,000 person-years, corresponding to an AAPC of 3.9%.^3^ In the United States, the age-adjusted incidence rate was 5.5 per 100,000 person-years between 2001 and 2021, with an AAPC of 2.5%.^20^ In contrast, global incidence declined from 9.0 per 100,000 person-years in 1990 to 6.5 per 100,000 person-years in 2019, largely driven by reductions in East Asia following effective HBV vaccination programs.^4^^,^^21^

ALD was the most frequent etiology in our cohort, followed by MASLD. Notably, nearly one-fifth of patients had no recorded underlying risk factor for chronic liver disease, and the etiology was unknown in one-third of the cohort. Globally, HBV and HCV remain the dominant causes of HCC, although non-viral etiologies are increasing.^22^^,^^23^ The rising incidence of MASLD-related HCC in our cohort aligns with this shift. Nevertheless, some misclassification is likely. Among patients registered without an apparent risk factor for chronic liver disease, 40.7% had at least one cardiometabolic risk factor, suggesting that some may have had undiagnosed MASLD. Due to missing data on obesity, dyslipidemia, and hypertension, the true proportion of MASLD-related HCC may have been underestimated. Similar patterns have been reported previously.^24^

MASLD-related HCC was associated with better overall survival than ALD-related HCC and HCC with unknown etiology. This improved overall survival probability persisted after adjusting for treatment allocation, age, sex, CP score, tumor diameter, number of nodules, and the presence of distant metastases. Overall, curative-intent treatment was the strongest predictor of overall mortality, followed by other tumor-directed treatment, the presence of distant metastases, and CP-C.

The absence of cirrhosis was noted in a substantial proportion of patients. Notably, in the final 3 years of our study period, more than 30% of patients were recorded as not having cirrhosis, consistent with previous studies.[25], [26], [27] Patients without cirrhosis presented with larger tumor diameters and more frequently had unifocal disease, in line with earlier findings.^28^ Although larger tumors in this group may reflect the absence of surveillance, the proportion receiving curative-intent treatment did not differ between patients with and without cirrhosis in our cohort. The preserved liver function and unifocal nature of tumors in non-cirrhotic patients may partially explain their high eligibility for curative therapy despite later detection.

This study has several limitations inherent to its retrospective, registry-based design. A considerable proportion of patients had missing data, particularly regarding etiology and cirrhotic status. Patients with an unknown etiology generally presented with larger tumors, less frequently had solitary lesions, were less likely to receive tumor-directed treatment, and had poorer overall survival. This group likely represents patients presenting with more advanced disease, potentially resulting in less extensive diagnostic evaluation, especially in the context of a high comorbidity burden and limited treatment options. Consequently, survival estimates among patients with a documented etiology may be overly optimistic. Given the extent of missing data, comparisons between etiological subgroups should be interpreted with caution and primarily viewed as descriptive. In addition, the data captured by the NCR lack key tumor-specific or clinical variables, including alpha-fetoprotein levels, performance status, detailed comorbidity indices, and quantitatively assessed alcohol consumption. The absence of alcohol consumption data limits insight into potential overlap between MASLD and ALD, which may have led to misclassification. Furthermore, the recent nomenclature change from NAFLD to MASLD in 2023, accompanied by modified diagnostic criteria, may have introduced classification inconsistencies over time.^5^ Although registry data are systematically collected, some degree of misclassification cannot be excluded.

The survival analyses should be interpreted as strictly descriptive. Although multivariable Cox regression was performed to adjust for measured covariates, residual confounding is likely. Treatment allocation is strongly influenced by baseline disease severity, liver function, age, and comorbidity, factors that are incompletely captured in the registry. Therefore, the observed associations between treatment allocation and survival should not be interpreted as causal effects. Moreover, the analyses are susceptible to immortal time bias, potentially leading to an overestimation of survival differences between treatment groups. Given the multiple subgroup comparisons performed, the analyses were not designed for formal hypothesis testing. No correction for multiplicity was applied; therefore, the findings should be considered as descriptive patterns rather than confirmatory evidence. A key strength of this study is the use of a large, nationwide, unselected cohort, enabling detailed description of national temporal trends and clinical patterns of HCC.

In conclusion, this nationwide study of HCC in the Netherlands highlights a steadily increasing incidence rate over the past decade, with the most pronounced rise observed in MASLD-related HCC and in patients without a documented underlying risk factor for chronic liver disease. Overall survival remains poor, particularly among patients with an unknown etiology or cirrhotic status, who are likely not recognized as having underlying liver disease prior to HCC diagnosis and are therefore not enrolled in regular surveillance programs. These findings underscore the need for greater clinical awareness and earlier identification of underlying liver disease to facilitate timely detection, appropriate management, and ultimately improved long-term patient survival.

## Abbreviations

AAPC, average annual percentage change; ALD, alcohol-related liver disease; CVD, cardiovascular disease; CP, Child–Pugh; HBV, hepatitis B virus; HCC, hepatocellular carcinoma; HCV, hepatitis C virus; HR, hazard ratio; MASLD, metabolic dysfunction-associated liver disease; NCR, Netherlands Cancer Registry.

## Authors’ contributions

Conceptualization: KvS, KvE, JdV-G, HK, ET, JB, NHM, DS, MC, FvV, OD, JD, MT, AH, LvdG, RT. Data curation: KvS, LvdG. Formal analysis: KvS, LvdG. Funding acquisition: JdV-G, HK, ET, JB, NHM, DS, MC, FvV, OD, JD, MT, AH, LvdG, RT. Investigation: KvS, KvE, LvdG, RT. Methodology: KvS, KvE, LvdG, RT. Project administration: KvS, RT. Software: not applicable. Resources: LvdG. Supervision: JdV-G, HK, ET, JB, NHM, DS, MC, FvV, OD, JD, MT, AH, LvdG, RT. Validation: not applicable. Visualization: K*vs.* Writing – original draft: KvS, KvE, LvdG, RT. Writing – review & editing: KvS, KvE, JdV-G, HK, ET, JB, NHM, DS, MC, FvV, OD, JD, MT, AH, LvdG, RT.

## Data availability

Data that support the findings of this study originate from the Netherlands Cancer Registry (NCR), maintained by the Netherlands Comprehensive Cancer Organisiation (IKNL). These data are not publicly available due to privacy and confidentiality restrictions. Access can be requested via IKNL’s Data Request procedure (https://iknl.nl/en/ncr/apply-for-data).

## Financial support

This study was financially supported by the MDLS NLA2 grant (WO-21-06) and the MLDS NAFLD-NL grant (WOO 20-07). The funder had no role in the design, conduct, or reporting of the study.

## Conflicts of interest

No conflict of interest: KvS, KvE, JdV-G, HK, ET, JB, NHM, DS, MC, FvV, OD, JD, MT, AH, LvdG, RT.

Please refer to the accompanying ICMJE disclosure forms for further details.
