# Supplementary material for: Increasing incidence and changing trends in etiology & survival rates in HCC in a European cohort
Source: JHEP Rep. 2026 Jun 23;8(10):101924. doi: 10.1016/j.jhepr.2026.101924 (PMC13545717; doi:10.1016/j.jhepr.2026.101924)
Supplement: Supplementary file 1 [file mmc1.pdf]

Increasing incidence and changing trends in etiology & survival rates in HCC in a European cohort

Koen C. van Son, Kirsi M.A. van Eekhout, Judith de Vos-Geelen, Heinz-Josef Klümpen, Eric T.T.L. Tjwa, Joep de Bruijne, Nadia Haj Mohammad, Dave Sprengers, Minneke J. Coenraad, Frederike G.I. van Vilsteren, Otto M. van Delden, Joost P.H. Drenth, Maarten E. Tushuizen, Adriaan G. Holleboom, Lydia G. van der Geest, R. Bart Takkenberg for the Dutch Hepatocellular & Cholangio Carcinoma Group (DHCG)

Table S1 2
Table S2 4
Table S3 5
Table S4 7
Table S5 9

Supplementary tables

Included morphology codes			
8000	Neoplasm, not otherwise specified (no microscopic verification)		
8001	Malignant tumor cells		
8170	HCC not further specified		
8171	Fibrolamellar HCC		
8172	Scirrhous HCC		
8173	Sarcomatoid HCC		
8174	Clear cell HCC		
8175	Pleomorphic HCC		
Included NCR variables			
Variable	NCR variable	Study variable	
Year of diagnosis	Year	Year	
Sex	Men	Men	
	Women	Women	
T2DM	Yes	Yes	
	No	No	
	Unknown or no available data	Unknown	
CVD	Myocardial infarction/CVD/congestive heart disease or peripheral heart disease present	Yes	
	Myocardial infarction/CVD/congestive heart disease and peripheral heart disease absent	No	
	Myocardial infarction/CVD/congestive heart disease and peripheral heart disease unknown or no available data	Unknown	
Age at diagnosis	Year	Year	
Etiology			Hierarchical order
	Hepatitis B	HBV	1
	Hepatitis C	HCV	2
	Alcoholic cirrhosis	ALD	3
	NAFLD/NASH	MASLD	4
	Auto-immune hepatitis/primary biliary cirrhosis	Other	5
	Hemochromatosis/M. Wilson/porphyria	Other	6
	No risk factor	No risk factor	7
	Liver cirrhosis with unknown etiology/unknown	Unknown	NA

	cause/cryptogenic cirrhosis or no available data		
Cirrhosis	Liver cirrhosis, no CP score	Yes	
	CP-A, CP-B, or CP-C	Yes	
	CP-0	No	
	Unknown or no available data	Unknown	
CP score	0	No cirrhosis	
	Liver cirrhosis, no CP score	Cirrhosis, CP unknown	
	Unknown or no available data	Unknown	
	A	A	
	B	B	
	C	C	
Tumor diameter	mm	mm	
Number of nodules	1	1	
	2	2	
	3	3	
	Multifocal/diffuse	Multifocal/diffuse	
	No available data	Unknown	
Distant metastasis	Yes	Yes	
	No	No	
	Unknown	Unknown	
Treatment	Resection, primary tumor destruction using RFA or MWA, or liver transplantation	Curative-intent	
	Tumor-directed tumor other than resection, primary tumor destruction using RFA or MWA, or liver transplantation	Other tumor-directed	
	No tumor-directed treatment	No tumor-directed	

Table S1: Overview of the included data. HCC, hepatocellular carcinoma; NCR, Netherlands Cancer Registry; T2DM, type 2 diabetes mellitus; CVD, cardiovascular disease; NAFLD/NASH, non-alcoholic fatty liver disease/non-alcoholic steatohepatitis; MASLD, metabolic dysfunction-associated steatotic liver disease; ALD, alcoholic liver disease; HBV, hepatitis B virus; HCV, hepatitis C virus; CP, Child-Pugh; RFA, radiofrequency ablation; MWA, microwave ablation.

	Age-adjusted incidence rate (per 100.000 person-years)									AAPC (95% CI)
	2014	2015	2016	2017	2018	2019	2020	2021	2022	
HCC	3.53	3.93	3.70	4.16	4.40	4.37	4.27	4.61	4.12	2.3 (0.9-3.7)
Stratified by etiology										
MASLD	0.29	0.25	0.29	0.37	0.61	0.48	0.60	0.60	0.48	8.3 (2.9-13.7)
ALD	0.78	0.75	0.62	0.78	0.87	0.95	0.90	0.92	0.83	2.1 (-1.3-5.5)
HBV	0.37	0.35	0.21	0.33	0.36	0.33	0.21	0.24	0.25	-4.5 (-11.4-2.4)
HCV	0.31	0.41	0.27	0.32	0.37	0.32	0.28	0.29	0.27	-2.5 (-6.7-1.6)
Other	0.11	0.12	0.07	0.12	0.10	0.13	0.17	0.12	0.11	1.9 (-5.6-9.4)
No risk factor	0.55	0.80	0.53	0.65	0.62	0.71	0.87	0.97	0.99	6.7 (2.4-10.9)
Unknown	1.12	1.24	1.70	1.58	1.47	1.45	1.24	1.47	1.19	1.7 (-0.6-4.1)

Table S2: Age-adjusted incidence rate and average annual percentage change of HCC in the Netherlands between 2014 and 2022 stratified by etiology (n=6,526). AAPC = average annual percent change; HCC = hepatocellular carcinoma; MASLD = metabolic dysfunction-associated steatotic liver disease; ALD = alcohol-related liver disease, HBV = hepatitis B virus, HCV = hepatitis C virus.

		Etiology							
Variable		MASLD	ALD	HBV	HCV	Other	No risk factor	Unknown	p-value
n		711	1,322	466	508	186	1,175	2,158	
Year of diagnosis, n (%)	2014	46 (6.5)	128 (9.7)	63 (13.5)	53 (10.4)	18 (9.7)	87 (7.4)	175 (8.1)	<0.001*
	2015	41 (5.8)	125 (9.5)	60 (12.9)	70 (13.8)	21 (11.3)	129 (11.0)	200 (9.3)	
	2016	50 (7.0)	107 (8.1)	36 (7.7)	48 (9.4)	12 (6.5)	88 (7.5)	283 (13.1)	
	2017	64 (9.0)	137 (10.4)	58 (12.4)	57 (11.2)	20 (10.8)	110 (9.4)	268 (12.4)	
	2018	107 (15.0)	154 (11.6)	63 (13.5)	66 (13.0)	18 (9.7)	108 (9.2)	251 (11.6)	
	2019	88 (12.4)	174 (13.2)	58 (12.4)	58 (11.4)	23 (12.4)	126 (10.7)	259 (12.0)	
	2020	110 (15.5)	165 (12.5)	38 (8.2)	51 (10.0)	31 (16.7)	158 (13.4)	224 (10.4)	
	2021	113 (15.9)	174 (13.2)	44 (9.4)	54 (10.6)	22 (11.8)	179 (15.2)	272 (12.6)	
	2022	92 (12.9)	158 (12.0)	46 (9.9)	51 (10.0)	21 (11.3)	190 (16.2)	226 (10.5)	
Sex, n men (%)		504 (70.9)	1,120 (84.7)	401 (86.1)	387 (76.2)	120 (64.5)	856 (72.9)	1,513 (70.1)	<0.001*
T2DM, n (%)	Yes	353 (49.6)	335 (25.3)	78 (16.7)	74 (14.6)	40 (21.5)	361 (30.7)	553 (25.6)	<0.001*
	No	154 (21.7)	541 (40.9)	191 (41.0)	237 (46.7)	81 (43.5)	471 (40.1)	705 (32.7)	
	Unknown	204 (28.7)	446 (33.7)	197 (42.3)	197 (38.8)	65 (34.9)	343 (29.2)	900 (41.7)	
CVD, n (%)	Yes	132 (18.6)	181 (13.7)	41 (8.8)	46 (9.1)	26 (14.0)	254 (21.6)	373 (17.3)	<0.001*
	No	375 (52.7)	695 (52.6)	228 (48.9)	265 (52.2)	95 (51.1)	578 (49.2)	885 (41.0)	
	Unknown	204 (28.7)	446 (33.7)	197 (42.3)	197 (38.8)	65 (34.9)	343 (29.2)	900 (41.7)	
Age at diagnosis, years (mean (SD))		70.0 (8.2)	68.4 (7.7)	61.1 (12.4)	63.0 (8.2)	67.2 (12.2)	71.9 (11.4)	73.0 (10.4)	<0.001**
Cirrhosis, n (%)	Yes	538 (75.7)	1,106 (83.7)	274 (58.8)	387 (76.2)	118 (63.4)	83 (7.1)	717 (33.2)	<0.001*
	No	98 (13.8)	37 (2.8)	110 (23.6)	54 (10.6)	37 (19.9)	971 (82.6)	369 (17.1)	
	Unknown	75 (10.5)	179 (13.5)	82 (17.6)	67 (13.2)	31 (16.7)	121 (10.3)	1,072 (49.7)	
Child-Pugh score, n (%)	A	359 (50.5)	563 (42.6)	182 (39.1)	263 (51.8)	73 (39.2)	52 (4.4)	358 (16.6)	<0.001*
	B	135 (19.0)	345 (26.1)	58 (12.4)	78 (15.4)	33 (17.7)	16 (1.4)	199 (9.2)	
	C	23 (3.2)	119 (9.0)	18 (3.9)	29 (5.7)	6 (3.2)	2 (0.2)	53 (2.5)	
	Cirrhosis, CP score unknown	21 (3.0)	79 (6.0)	16 (3.4)	17 (3.3)	6 (3.2)	13 (1.1)	107 (5.0)	
	No cirrhosis	98 (13.8)	37 (2.8)	110 (23.6)	54 (10.6)	27 (19.9(	971 (82.6)	369 (17.1)	
	Unknown cirrhotic status	75 (10.5)	179 (13.5)	82 (17.6)	67 (13.2)	31 (16.7)	121 (10.3)	1,072 (49.7)	

Tumor diameter, mm (median (IQR))		34 (23, 58)	36 (23, 60)	45 (25, 88)	34 (22, 60)	40 (27, 76)	85 (50, 130)	67 (38, 102)	<0.001***
Number of nodules, n (%)	1	359 (50.5)	539 (40.8)	235 (50.4)	237 (46.7)	98 (52.7)	623 (53.0)	767 (35.5)	<0.001*
	2	123 (17.3)	225 (17.0)	69 (14.8)	67 (13.2)	33 (17.7)	128 (10.9)	209 (9.7)	
	3	53 (7.5)	94 (7.1)	25 (5.4)	47 (9.3)	14 (7.5)	54 (4.6)	103 (4.8)	
	Multifocal/ diffuse	159 (22.4)	418 (31.6)	130 (27.9)	145 (28.5)	36 (19.4)	342 (29.1)	708 (32.8)	
	Unknown	17 (2.4)	46 (3.5)	7 (1.5)	12 (2.4)	5 (2.7)	28 (2.4)	371 (17.2)	
Distant metastasis, n (%)	Yes	63 (8.9)	171 (12.9)	93 (20.0)	58 (11.4)	22 (11.8)	281 (23.9)	575 (26.6)	<0.001*
		643 (90.4)	1,131 (85.6)	367 (78.8)	445 (87.6)	162 (87.1)	875 (74.5)	1,474 (68.3)	
	Unknown	5 (0.7)	20 (1.5)	6 (1.3)	5 (1.0)	2 (1.1)	19 (1.6)	109 (5.1)	
Treatment, n (%)	Curative-intent	278 (39.1)	384 (29.0)	174 (37.3)	196 (38.6)	66 (35.5)	331 (28.2)	333 (15.4)	<0.001*
	Other tumor-directed	244 (34.3)	383 (29.0)	141 (30.3)	129 (25.4)	50 (26.9)	351 (29.9)	421 (19.5)	
	No tumor-directed	189 (26.6)	555 (42.0)	151 (32.4)	183 (36.0)	70 (37.6)	493 (42.0)	1,404 (65.1)	

Table S3: Patient characteristics stratified by etiology (n=6,526). SD = standard deviation; IQR = interquartile range; T2DM = type 2 diabetes mellitus; CVD = cardiovascular disease; MASLD = metabolic-dysfunction associated steatotic liver disease, ALD = alcohol-related liver disease, HBV = hepatitis B virus; HCV = hepatitis C virus; CP, Child-Pugh. * Chi-square test; ** One-way ANOVA; *** Kruskal-Wallis test.

		Presence of cirrhosis				
Variable		Yes	No	p-value (Yes vs. No)	Unknown	p-value (Yes vs. No vs. Unknown)
n		3,223	1,676		1,627	
Year of diagnosis, n (%)	2014	246 (7.6)	103 (6.1)	<0.001*	221 (13.6)	<0.001*
	2015	267 (8.3)	165 (9.8)		214 (13.2)	
	2016	263 (8.2)	101 (6.0)		240 (14.8)	
	2017	322 (10.0)	152 (9.1)		240 (14.8)	
	2018	371 (11.5)	165 (9.8)		231 (14.2)	
	2019	418 (13.0)	179 (10.7)		189 (11.6)	
	2020	433 (13.4)	244 (14.6)		100 (6.1)	
	2021	454 (14.1)	298 (17.8)		106 (6.5)	
	2022	449 (13.9)	269 (16.1)		66 (4.1)	
Sex, n men (%)		2,522 (78.3)	1,223 (73.0)	<0.001*	1,156 (71.1)	<0.001*
T2DM, n (%)	Yes	1,011 (31.4)	489 (29.2)	<0.001*	294 (18.1)	0.006*
	No	1,252 (38.8)	730 (43.6)		398 (24.5)	
	Unknown	960 (29.8)	457 (27.3)		935 (57.5)	
CVD, n (%)	Yes	500 (15.5)	359 (21.4)	<0.001*	194 (11.9)	<0.001*
	No	1,763 (54.7)	860 (51.3)		498 (30.6)	
	Unknown	960 (29.8)	457 (27.3)		934 (57.5)	
Age at diagnosis, years (mean (SD))		1,763 (54.7)	860 (51.3)	<0.001**	498 (30.6)	<0.001**
Etiology, n (%)	MASLD	538 (16.7)	98 (5.8)	<0.001*	75 (4.6)	<0.001*
	ALD	1,106 (16.7)	37 (2.2)		179 (11.0)	
	HBV	274 (8.5)	110 (6.6)		82 (5.0)	
	HCV	387 (12.0)	54 (3.2)		67 (4.1)	
	Other	118 (3.7)	37 (2.2)		31 (1.9)	
	No risk factor	83 (2.6)	971 (57.9)		121 (7.4)	
	Unknown	717 (22.2)	369 (22.0)		1,072 (65.9)	
Tumor diameter, mm (median (IQR))		36 (23, 62)	80 (46, 122)	<0.001***	70 (36, 100)	<0.001***
Number of nodules, n (%)	1	1,391 (43.2)	923 (55.1)	<0.001*	544 (33.4)	<0.001*
	2	528 (16.4)	171 (10.2)		155 (9.5)	

	3	255 (7.9)	81 (4.8)		54 (3.3)	
	Multifocal/ diffuse	966 (30.0)	467 (27.9)		505 (31.0)	
	Unknown	83 (2.6)	34 (2.0)		369 (22.7)	
Distant metastasis, n (%)	Yes	431 (13.4)	379 (22.6)	<0.001*	453 (27.8)	<0.001*
	No	2,757 (85.5)	1,266 (75.5)		1,074 (66.0)	
	Unknown	35 (1.1)	31 (1.8)		100 (6.1)	
Treatment, n (%)	Curative-intent	1,002 (31.1)	505 (30.1)	p=0.004*	255 (15.7)	<0.001*
	Other tumor-directed	1,030 (32.0)	475 (28.3)		214 (13.2)	
	No tumor-directed	1,191 (37.0)	696 (41.5)		1,158 (71.2)	

Table S4: Patient characteristics stratified by cirrhotic status (n=6,526). SD = standard deviation; IQR = interquartile range; T2DM = type 2 diabetes mellitus; CVD = cardiovascular disease; MASLD = metabolic-dysfunction associated steatotic liver disease, ALD = alcohol-related liver disease, HBV = hepatitis B virus; HCV = hepatitis C virus. * Chi-square test; ** Unpaired t-test; *** Mann-Whitney U-test.

	1-year (%)	3-year (%)	5-year (%)	Log-rank test
Overall	46.5	26.1	18.8	-
Etiology				
MASLD	65.3	37.9	24.3	Ref.
ALD	50.0	24.7	16.1	<0.001
HBV	54.6	37.5	30.6	0.626
HCV	62.0	39.7	31.1	0.784
Other	56.8	33.1	25.6	0.194
No risk factor	48.8	28.9	21.9	<0.001
Unknown	30.7	15.5	10.6	<0.001
Child-Pugh score				
No cirrhosis	49.7	30.6	23.8	Ref.
A	69.8	40.5	27.0	<0.001
B	37.4	17.3	12.4	<0.001
C	17.9	9.7	8.6	<0.001
Cirrhosis, CP score unknown	35.5	18.7	14.6	<0.001
Unknown cirrhotic status	27.8	13.9	10.2	<0.001

Table S5: 1-, 3- and 5-year overall survival rate for the entire study population (n=6,526), and stratified for etiology (n=6,526) and Child-Pugh score (n=6,526) using long-rank test. MASLD = metabolic-dysfunction associated steatotic liver disease, ALD = alcohol-related liver disease, HBV = hepatitis B virus; HCV = hepatitis C virus.
