# Supplementary material for: Increasing incidence and changing trends in etiology & survival rates in HCC in a European cohort
Source: JHEP Rep. 2026 Jun 23;8(10):101924. doi: 10.1016/j.jhepr.2026.101924 (PMC13545717; doi:10.1016/j.jhepr.2026.101924)
Supplement: Supplementary file 3 [file mmc4.pdf]

Research article

JHEP Reports

Increasing incidence and changing trends in etiology & survival rates in HCC in a European cohort

Authors

Koen C. van Son, Kirsi M.A. van Eekhout, Judith de Vos-Geelen, …, Adriaan G. Holleboom, Lydia G. van der Geest, R. Bart Takkenberg

Correspondence

k.c.vanson@amsterdamumc.nl (K.C. van Son).

Graphical abstract

Incidence rate

- **6,526 consecutive patients** with newly diagnosed HCC in the Netherlands Cancer Registry (NCR) (2014-2021)

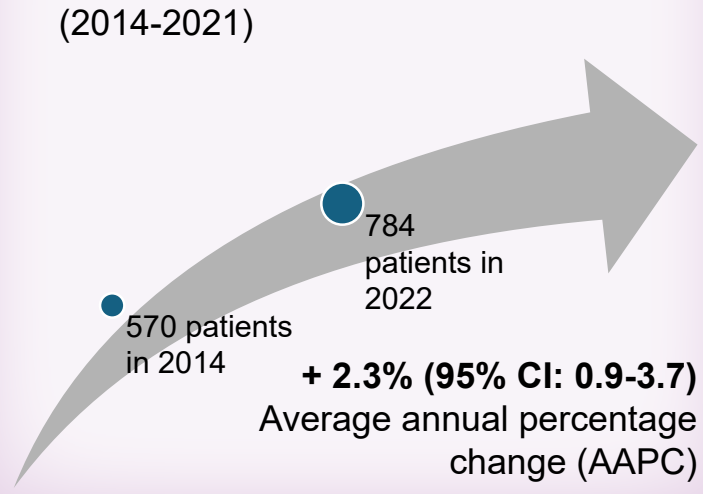

Changing HCC etiologies

	AAPC (95% CI)
HCC	+2.3 (0.9-3.7)
Stratified by etiology	
MASLD	+8.3 (2.9-13.7)
ALD	+2.1 (-1.3-5.5)
HBV	-4.5 (-11.4-2.4)
HCV	-2.5 (-6.7-1.6)
Other	+1.9 (-5.6-9.4)
No risk factor	+6.7 (2.4-10.9)
Unknown	+1.7 (-0.6-4.1)

Table 1: Average annual percentage change (AAPC) of HCC stratified by etiology

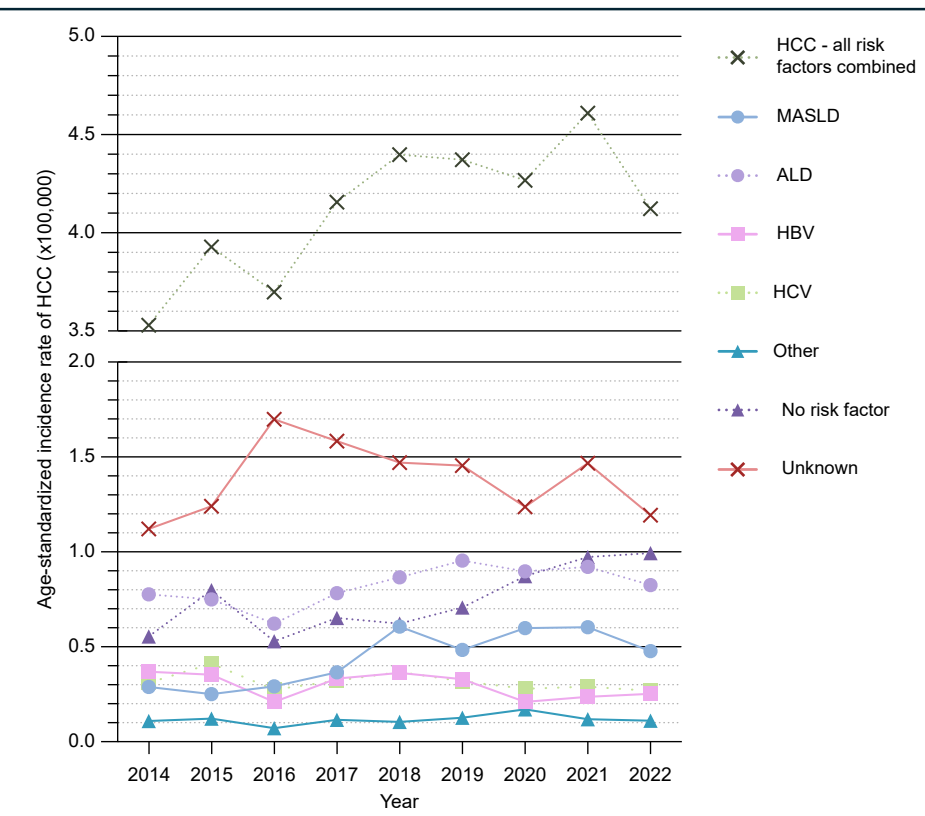

Conclusions

- Sustained increase in HCC incidence rates in the Netherlands
- Significant rise in **MASLD-related HCC** and HCC with no registered risk factor of chronic liver disease
- Overall survival rates remain poor
- Findings underscore the need for **increased clinical awareness** and further investigation into **surveillance strategies**

Survival rates

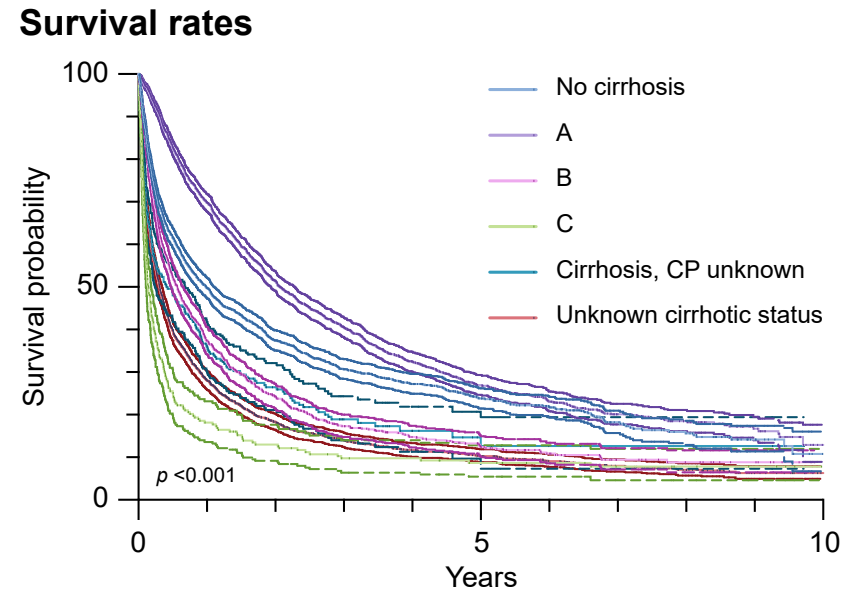

- ALD-related HCC had worse overall survival probability than MASLD-related HCC (HR: 1.21 (95% CI: 1.08-1.35))
- **Curative-intent treatment allocation** had the most profound effect on overall survival probability (HR: 0.17 (95% CI: 0.15-0.19))
- followed by **other tumor-directed treatment** (HR: 0.37 (95% CI: 0.34-0.40))
- and the **presence of distant metastases** (HR: 1.99 (95% CI: 1.82-2.15))

Highlights:

- Age-adjusted HCC incidence in the Netherlands rose by 2.3% annually (2014-2022).
- MASLD-related HCC increased almost 10% annually over the same time period.
- While overall survival was poor, MASLD-related HCC was associated with better survival probability than ALD-related HCC.
- Treatment was the strongest predictor of mortality, followed by distant metastases.

Impact and implications:

Hepatocellular carcinoma (HCC) represents a major global health problem. However, comprehensive data on longitudinal changes from nationwide cohorts remain limited. This study demonstrates that the incidence rate of HCC is increasing in the Netherlands. Furthermore, it identifies treatment allocation, the presence of distant metastases, Child-Pugh C, and multifocal disease as key predictors of overall mortality. Overall survival remains poor, particularly among patients with no registered risk factor for chronic liver disease or those with unknown cirrhotic status. These findings underscore the need for increased clinical awareness and early diagnostic evaluation of underlying liver disease to facilitate timely detection, appropriate management, and improved clinical outcomes.

https://doi.org/10.1016/j.jhepr.2026.101924

© 2026 The Author(s). Published by Elsevier B.V. on behalf of European Association for the Study of the Liver (EASL). This is an open access article under the CC BY license (http://creativecommons.org/licenses/by/4.0/).

JHEP|Reports

Increasing incidence and changing trends in etiology & survival rates in HCC in a European cohort

Koen C. van Son[1,2,3,*], Kirsi M.A. van Eekhout[1,2,3], Judith de Vos-Geelen[4], Heinz-Josef Klümpen[5], Eric T.T.L. Tjwa[6], Joep de Bruijne[7], Nadia Haj Mohammad[8], Dave Sprengers[9], Minneke J. Coenraad[10], Frederike G.I. van Vilsteren[11], Otto M. van Delden[12], Joost P.H. Drenth[2], Maarten E. Tushuizen[10], Adriaan G. Holleboom[1], Lydia G. van der Geest[13], R. Bart Takkenberg[2], for the Dutch Hepatocellular & Cholangio Carcinoma Group (DHCG)

JHEP Reports **2026**. vol. 8 | 1–8

Check for updates

Background & Aims: Hepatocellular carcinoma (HCC) represents a major global health burden. While incidence rates are declining in some regions, they are rising in the Netherlands, traditionally a low-endemic country for HCC. Despite these trends, comprehensive data on longitudinal changes in etiology, tumor characteristics, and overall survival remain scarce.

Methods: Consecutive patients registered in the Netherlands Cancer Registry with newly diagnosed HCC were included (2014-2022). Average annual percentage change (AAPC) was calculated to assess trends in age-adjusted incidence rates. Cox regression analyses were performed to determine risk factors for overall mortality.

Results: Between January 2014 and December 2022, 6,526 patients were diagnosed with HCC in the Netherlands. The age-adjusted incidence rate of HCC rose from 3.5 to 4.1 per 100,000 person-years, reflecting an AAPC of 2.3% (95% CI 0.9-3.7). While alcohol-related liver disease (ALD) was the most common etiology of HCC, metabolic dysfunction-associated steatotic liver disease (MASLD)-related HCC had the highest AAPC at 8.3% (95% CI 2.9-13.7). 1-, 3- and 5-year survival rates were 46.5%, 26.1% and 18.8%, respectively. ALD-related HCC was associated with worse overall survival probability than MASLD-related HCC (hazard ratio [HR] 1.21; 95% CI 1.08-1.35). Curative-intent treatment allocation had the most profound effect on overall survival probability (HR 0.17; 95% CI 0.15-0.19), followed by other tumor-directed treatment (HR 0.37; 95% CI 0.34-0.40), and the presence of distant metastases (HR 1.99; 95% CI 1.83-2.15).

Conclusion: This comprehensive nationwide cohort study demonstrates a sustained increase in HCC incidence rates in the Netherlands, with a significant rise in MASLD-related HCC. Overall survival remains poor. These findings underscore the need for increased clinical awareness and further investigation into surveillance strategies.

© 2026 The Author(s). Published by Elsevier B.V. on behalf of European Association for the Study of the Liver (EASL). This is an open access article under the CC BY license (http://creativecommons.org/licenses/by/4.0/).

Introduction

Primary liver cancer is the eighth most prevalent malignancy globally and the third leading cause of cancer-related mortality.[1] Hepatocellular carcinoma (HCC) accounts for approximately 90% of all primary liver cancers and represents a major global health burden.[2] While HCC incidence rates are declining in certain regions, they are increasing in traditionally low endemic countries such as the Netherlands.[3,4] Despite these trends, comprehensive data on longitudinal changes in etiology, tumor characteristics, and overall survival among patients with HCC remain scarce.

The primary risk factor for HCC is chronic liver disease, most notably hepatitis B virus (HBV) and hepatitis C virus (HCV) infections, alcohol-related liver disease (ALD), and metabolic dysfunction-associated steatotic liver disease (MASLD).[5] Globally, HBV and HCV remain the most common etiologies for HCC. However, the widespread adoption of HBV vaccination and improved antiviral therapies for HCV have led to a decline in virus-associated HCC incidence.[6] Conversely, the incidence and prevalence of MASLD are on the rise, currently affecting approximately 30% of adults worldwide,[7] driven by the increasing prevalence of type 2 diabetes mellitus and obesity.[8] As a consequence, the incidence of MASLD-related HCC is growing, with projections suggesting increases ranging from 44% in Japan to 122% in the United States by 2030.[9]

Despite these shifts, the literature on overall survival rates of patients with MASLD-related HCC remains conflicting. Some studies report poorer overall survival for patients with MASLD-related HCC compared to those with other etiologies,[10–12] while other studies find no significant differences.[13,14] Additionally, a significant clinical challenge with MASLD-related HCC is its development in the absence of cirrhosis in approximately 40% of cases. Since surveillance programs

* Corresponding author. Address: Department of Vascular Medicine, Amsterdam UMC, location Meibergdreef, room M0-118, Meibergdreef 9, 1105 AZ Amsterdam, The Netherlands. Tel.: +31627150710.
E-mail address: k.c.vanson@amsterdamumc.nl (K.C. van Son).
https://doi.org/10.1016/j.jhepr.2026.101924

typically rely on the presence of cirrhosis, early detection of HCC in this population is often hampered.[15]

Most existing data are derived from specialized centers or specific cancer registries, which may not fully reflect routine clinical practice. Therefore, this study aimed to investigate the incidence, etiology, tumor characteristics, and overall survival of patients with HCC using data from the nationwide Netherlands Cancer Registry (NCR) between 2014 and 2022.

Patients and methods

Study population and data collection

All patients registered with HCC in the NCR from January 2014 to December 2022 were included. The NCR is a nationwide population-based cancer registry that covers the entire Dutch population of approximately 18 million people. Cancer diagnoses were notified to the NCR from the Dutch Nationwide Pathology Archives (PALGA) and the Dutch National Hospital Care Registry (*i.e.* hospital discharges and outpatient visits). All cases were confirmed through verification of patient records in all Dutch hospitals by trained registration clerks approximately 9 months after diagnosis. Vital status was updated annually by linkage with the nationwide municipal administrative database. HCC was defined according to the ICD-O-3 (International Classification of Diseases for Oncology Third Edition) topography C22.0 combined with morphology codes (Table S1).[16] Overall survival was defined as the time from diagnosis to death (any cause) or the end of follow-up (emigration or 1 February 2024). When multiple risk factors for HCC were present, a hierarchical order was applied to determine which etiology was recorded (Table S1). The presence of cirrhosis was determined based on pathological reports or, if pathology was unavailable, on radiological assessment. If reports did not contain clear information about its presence or absence, registration clerks recorded ‘unknown’. Curative-intent treatment was defined as resection, primary tumor destruction using radiofrequency ablation or microwave ablation, or liver transplantation. Other tumor-directed treatment was defined as any tumor-directed therapy that did not meet the aforementioned curative criteria (Table S1). All data were pseudoanonymized. The study proposal was evaluated by the Dutch Hepatocellular & Cholangiocarcinoma Group and the Privacy Review Board of the NCR (K22.269). The Institutional Medical Ethics Committee of the Amsterdam University Medical Center waived the need for ethical approval and individual informed consent. The reporting of this study followed the STROBE (Strengthening the Reporting of Observational Studies in Epidemiology) guidelines.[17]

Statistical analysis

Statistical analyses were performed using R (version 4.2.1; R Foundation for Statistical Computing, Vienna, Austria). p values <0.05 were considered statistically significant. Patient characteristics were expressed as frequencies with percentages, means (±SD) or medians (IQR), depending on the data distribution. Data were stratified by year of diagnosis, etiology (MASLD, ALD, HBV, HCV, other (auto-immune hepatitis, primary biliary cirrhosis, hemochromatosis, Wilson’s disease, porphyria), no risk factor for chronic liver disease and unknown), and presence of cirrhosis (yes, no, unknown) (Table S1). Age-standardized incidence rates per 100,000 person-years were calculated using the revised European Standard Population.[18] Trends in these rates were analyzed using log-linear Joinpoint regression analysis and summarized using the average annual percentage change (AAPC) with a 95% CI. Boxplots and Kruskal-Wallis tests were utilized to

Table 1. Patient characteristics of NCR HCC cohort (N = 6,526).

	Total patient cohort (N = 6,526)
Year of diagnosis, n (%)	
2014	570 (8.7)
2015	646 (9.9)
2016	624 (9.6)
2017	714 (10.9)
2018	767 (11.8)
2019	786 (12.0)
2020	777 (11.9)
2021	858 (13.1)
2022	784 (12.0)
Sex, n men (%)	4,901 (75.1)
T2DM, n (%)	
Yes	1,794 (27.5)
No	2,380 (36.5)
Unknown	2,352 (36.0)
CVD, n (%)	
Yes	1,053 (16.1)
No	3,121 (47.8)
Unknown	2,352 (36.0)
Age at diagnosis, years (mean (SD))	69.8 (10.6)
Etiology, n (%)	
MASLD	711 (10.9)
ALD	1,322 (20.3)
HBV	466 (7.1)
HCV	508 (7.8)
Other	186 (2.9)
No risk factor	1,175 (18.0)
Unknown	2,158 (33.1)
Cirrhosis, n (%)	
Yes	3,223 (49.4)
No	1,676 (25.7)
Unknown	1,627 (24.9)
CP score, n (%)	
A	1,850 (28.3)
B	864 (13.2)
C	250 (3.8)
Cirrhosis, CP score unknown	259 (4.0)
No cirrhosis	1,676 (25.7)
Unknown cirrhotic status	1,627 (24.9)
Tumor diameter, mm (median (IQR))	50 (28, 91)
Number of nodules, n (%)	
1	2,858 (43.8)
2	854 (13.1)
3	390 (6.0)
Multifocal/diffuse	1,938 (29.7)
Unknown	486 (7.4)
Distant metastasis, n (%)	
Yes	1,263 (19.4)
No	5,097 (78.1)
Unknown	166 (2.5)
Treatment, n (%)	
Curative-intent	1,762 (27.0)
Other tumor-directed	1,719 (26.3)
No tumor-directed	3,045 (46.7)

ALD, alcohol-related liver disease; CP, Child–Pugh; CVD, cardiovascular disease; HBV, hepatitis B virus; HCV, hepatitis C virus; MASLD, metabolic dysfunction-associated steatotic liver disease; T2DM, type 2 diabetes mellitus.

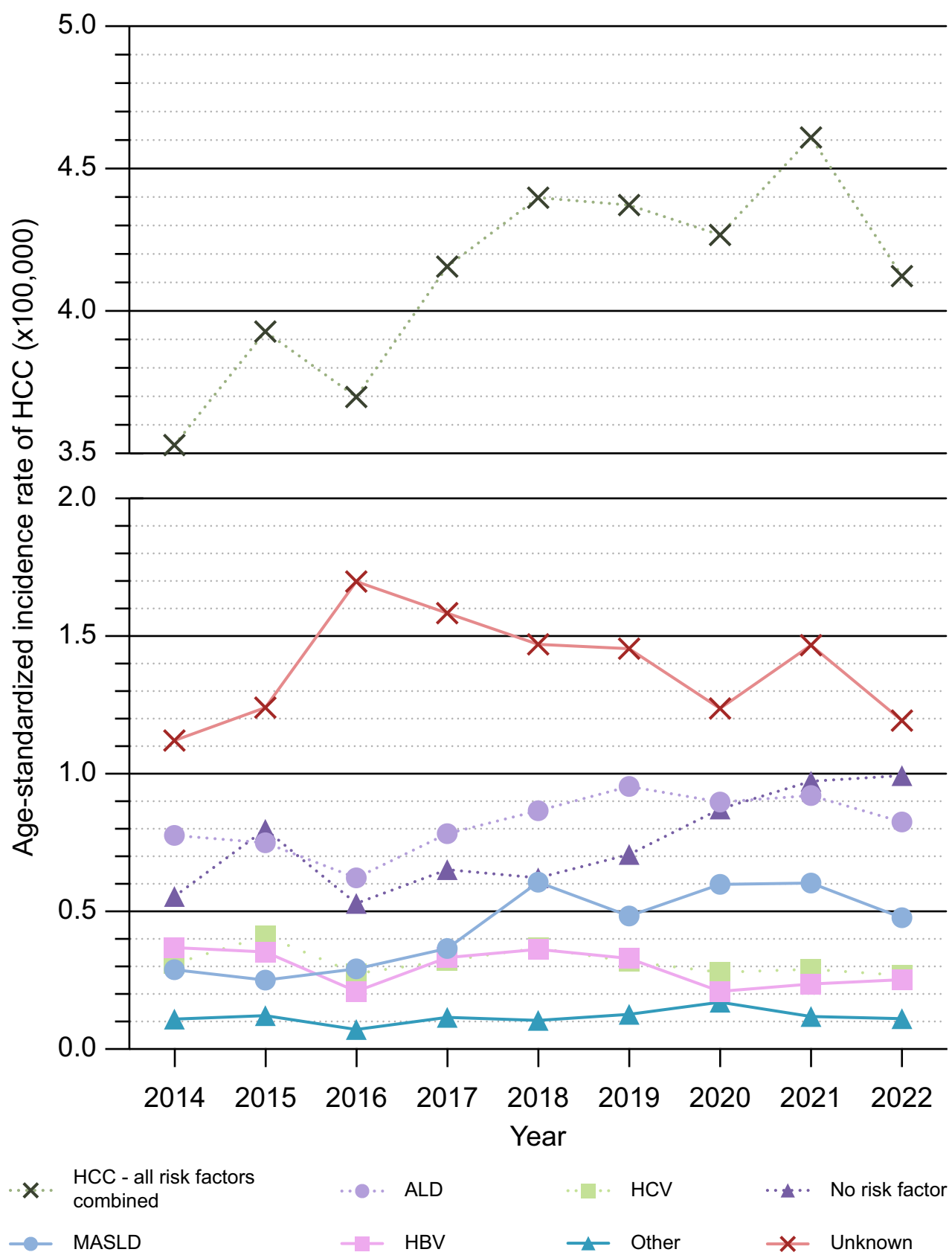

Fig. 1. Age-standardized incidence rate of HCC, and HCC stratified by etiology (N = 6,526). ALD, alcohol-related liver disease; HBV, hepatitis B virus; HCC, hepatocellular carcinoma; HCV, hepatitis C virus; MASLD, metabolic dysfunction-associated steatotic liver disease.

compare median tumor diameters stratified by risk factor and cirrhosis status (yes, no, unknown).

Kaplan-Meier survival curves, 1-, 3-, and 5-year survival rates, and log-rank tests were used to compare overall survival probability stratified by sex, etiology, and Child-Pugh (CP) score. Cox regression analyses were used to evaluate possible predictors of better survival, *e.g.* sex, age at diagnosis, etiology, CP score, tumor diameter, number of nodules, the presence of distant metastases, and treatment allocation Table 1.

Results

Between January 2014 and December 2022, 6,526 patients were diagnosed with HCC in the Netherlands. The annual number of HCC diagnoses increased from 570 in 2014 to 784 in 2022, with a peak of 858 diagnoses in 2021. The age-adjusted incidence rate rose from 3.5 to 4.1 per 100,000 person-years, corresponding to an AAPC of 2.3% (95% CI 0.9-3.7) (Fig. 1; Table 2). Mean age at diagnosis was 69.8 s (±10.6) years, and 75.1% of patients were men (Table 1).

ALD was the most common etiology of HCC (20.3%), followed by MASLD (10.9%), HCV (7.8%), HBV (7.1%), and other causes (2.9%). No underlying risk factor for chronic liver disease was found in 18.0% of patients, while etiology was unknown in 33.1% (Fig. 2A; Table 1). Fig. 1 shows age-adjusted HCC incidence rates stratified by etiology. MASLD-related HCC had the highest AAPC (8.3%; 95% CI 2.9-13.7) followed by HCC among patients registered as having no chronic liver disease (6.7%; 95% CI 2.4-10.9) (Tables 2 and S2). Other etiologies showed no significant trend over time (Fig. 1; Table 2). Patients without a registered risk factor for chronic liver disease and patients with unknown etiology presented with larger median tumor diameters compared to patients with any known etiology (Fig. 3A; Table S3). Patients with ALD-related HCC more often had multifocal (31.6% *vs.* 22.4%, p <0.001) and metastatic disease (12.9% *vs.* 8.9%, p = 0.007) than those with MASLD-related HCC (Table S3). In total, 27.0% of all patients received treatment with curative intent, 26.3% received other tumor-directed treatment, and 46.7% received no tumor-directed treatment (Table 1). Patients with MASLD-related HCC more often received curative-intent and other tumor-directed treatment than those with ALD-related HCC (39.1% *vs.* 29.0%, p <0.001 and 34.3% *vs.* 29.0%, p <0.001, respectively) (Table S3).

Cirrhosis was present in 3,223 (49.4%) patients, of whom 1,850 (57.4%) had CP-A, 864 (26.8%) had CP-B, and 250 (7.7%) had CP-C. Cirrhosis was absent in 1,676 (25.7%) patients, while cirrhotic status was unknown in 1,627 (24.9%) patients (Table 1). Among the 711 patients with MASLD-related HCC, cirrhosis was present in 538 (75.7%) patients and absent in 98 (13.8%) patients (Table S3; Fig. 2B). Patients with MASLD- and HCV-related HCC were more likely to have CP-A compared to those with ALD-related HCC. CP-C was more common among patients with ALD-related HCC than those with other etiologies (Fig. 2B; Table S3). The proportion of cases with unknown cirrhotic status decreased from over

Table 2. Average annual percentage change of HCC in the Netherlands between 2014 and 2022 stratified by etiology (N = 6,526).

	Age-adjusted incidence rate (per 100,000 person-years)	Age-adjusted incidence rate (per 100,000 person-years)	
	2014	2022	AAPC (95% CI)
HCC	3.53	4.12	2.3 (0.9-3.7)
**Stratified by etiology**			
MASLD	0.29	0.48	8.3 (2.9-13.7)
ALD	0.78	0.83	2.1 (-1.3 to 5.5)
HBV	0.37	0.25	-4.5 (-11.4 to 2.4)
HCV	0.31	0.27	-2.5 (-6.7 to 1.6)
Other	0.11	0.11	1.9 (-5.6 to 9.4)
No risk factor	0.55	0.99	6.7 (2.4-10.9)
Unknown	1.12	1.19	1.7 (-0.6 to 4.1)

AAPC, average annual percentage change; ALD, alcohol-related liver disease; HBV, hepatitis B virus; HCC, hepatocellular carcinoma; HCV, hepatitis C virus; MASLD, metabolic dysfunction-associated steatotic liver disease.

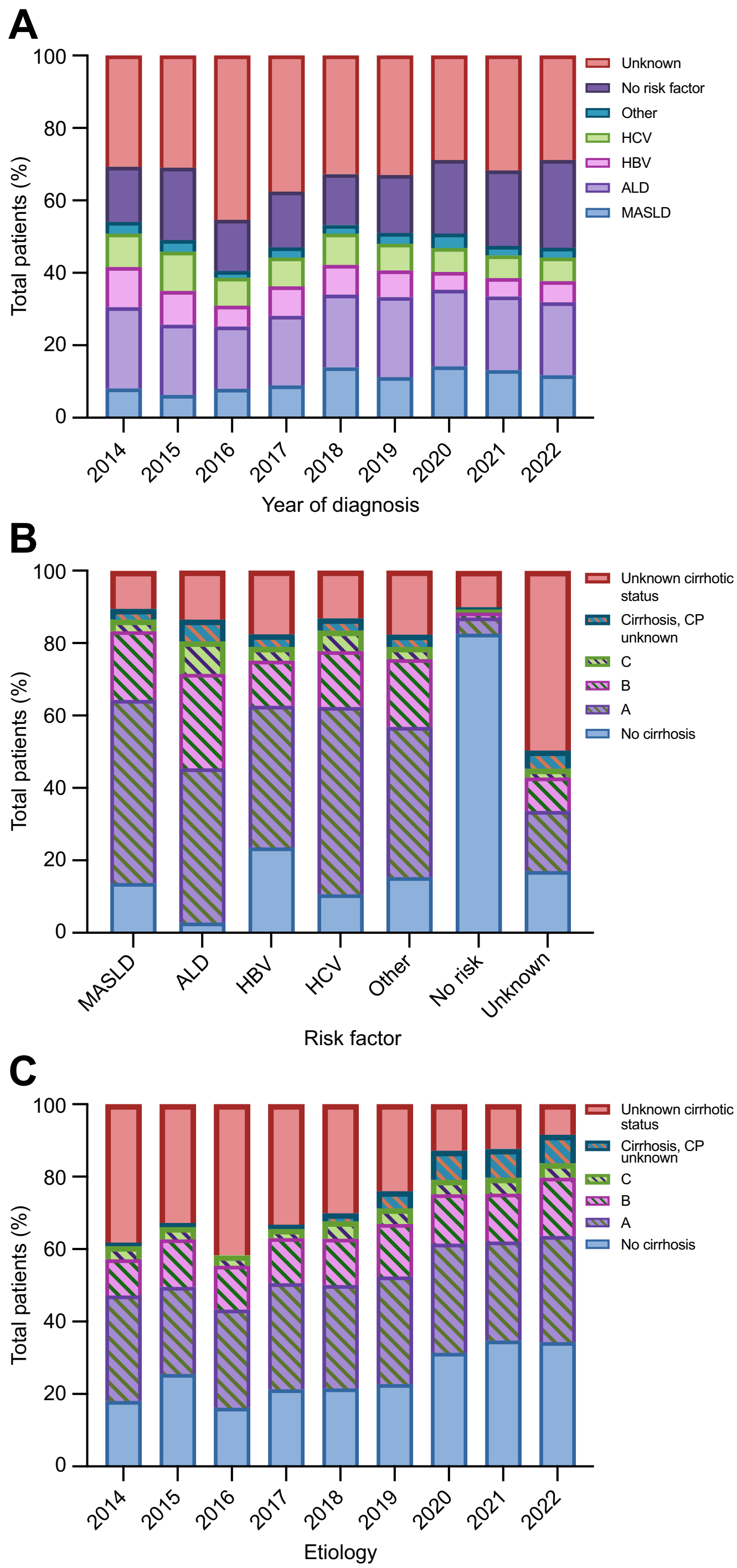

Fig. 2. Temporal trends in liver disease etiology and liver function at HCC diagnosis. Etiology by year of diagnosis (N = 6,526) (A), CP score stratified by etiology (N = 6,526) (B), and CP score per year of inclusion (N = 6,526) (C). Diagonal lines indicate the presence of cirrhosis. No risk = no risk factor for chronic liver disease. ALD, alcohol-related liver disease; CP, Child-Pugh; HBV, hepatitis B virus; HCC, hepatocellular carcinoma; HCV, hepatitis C virus; MASLD, metabolic dysfunction-associated steatotic liver disease.

30% to approximately 10% during the final 3 years of inclusion (Fig. 2C). Median tumor diameter was larger in patients without cirrhosis compared to those with cirrhosis (80 mm [IQR 46-122] *vss.* 36 mm [IQR 23-62], *p* <0.001) (Fig. 3B; Table S4). Patients without cirrhosis more frequently had distant metastases (22.6% *vs.* 13.4%, *p* <0.001) compared to those with cirrhosis. However, the proportion of patients receiving curative-intent treatment was similar between the two groups (30.1% *vs.* 31.1%, *p* = 0.700) (Table S4). Patients with cirrhosis more often received other tumor-directed treatment compared to those without cirrhosis (32.0% *vs.* 28.3%, *p* = 0.005).

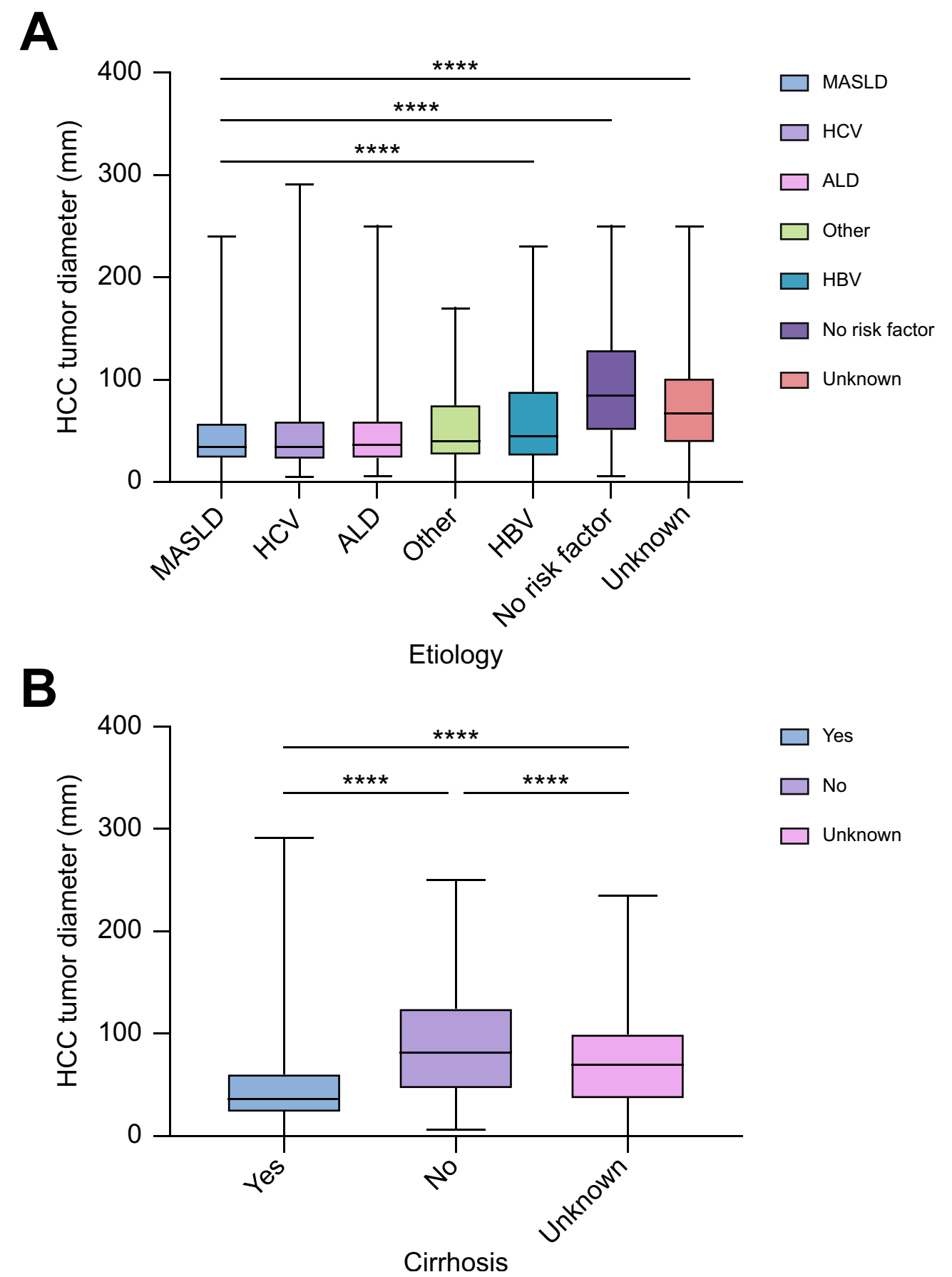

Fig. 3. Mean tumor diameter stratified by etiology and cirrhosic status. Mean tumor diameter (bars represent SD; bold lines inside the box plot represent medians) stratified by etiology (n = 5,490) (A), and by cirrhosis status (n = 5,490) (B). Levels of significance: ****p <0.001, ***p <0.005, **p <0.01, *p <0.05 (Kruskal-Wallis test). ALD, alcohol-related liver disease; HBV, hepatitis B virus; HCC, hepatocellular carcinoma; HCV, hepatitis C virus; MASLD, metabolic dysfunction-associated steatotic liver disease.

The 1-, 3-, and 5-year overall survival rates in the entire patient cohort were 46.5, 26.1 and 18.8%, respectively (Table S5). No difference in overall survival was observed between sexes (log-rank *p* = 0.530), nor was survival associated with year of diagnosis (log-rank *p* = 0.287) (Fig. 4A). One-, 3-, and 5-year overall survival rates were higher among patients with MASLD-related HCC (65.3%, 37.9% and 24.3%) compared to patients with ALD-related HCC (50.0%, 24.7% and 16.1%, log-rank *p* <0.001), patients registered as having no chronic liver disease (48.8%, 28.9% and 21.9%, log-rank *p* <0.001) and patients with unknown etiology (30.7%, 15.5% and 10.6%, log-rank *p* <0.001) (Fig. 4B; Table S5). When stratified by CP score, overall survival was poorest among those with CP-C (Fig. 4C; Table S5).

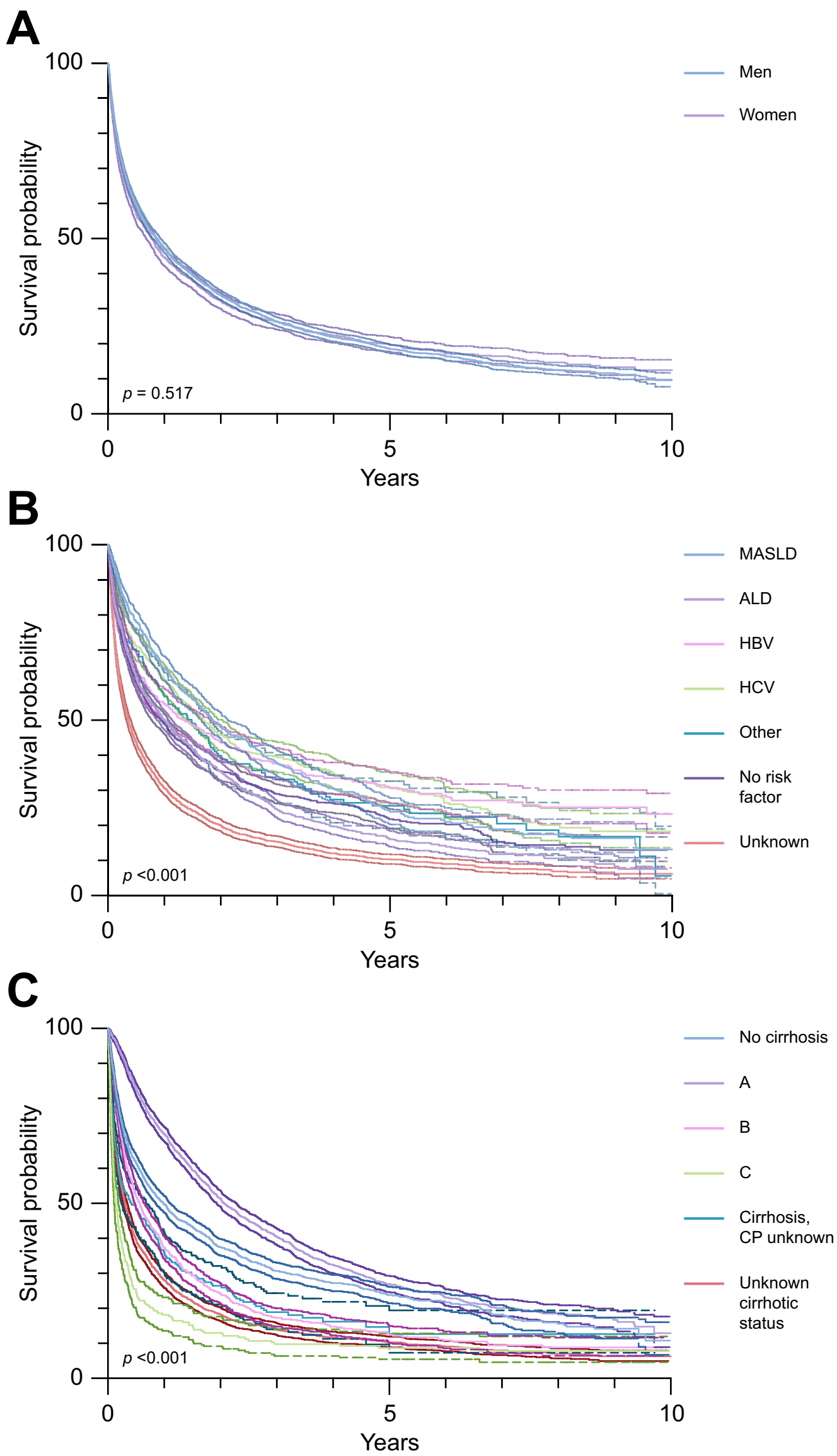

Fig. 4. Kaplan-Meier survival curve stratified by sex, etiology and CP score. Kaplan-Meier survival curve (bold lines represent survival probability; dashed lines represent 95% confidence intervals) stratified by sex (N = 6,526) (log-rank p = 0.493) (A), stratified by etiology (N = 6,526) (log-rank p <0.001) (B), and stratified by Child-Pugh score (N = 6,526) (log-rank p <0.001) (C). ALD, alcohol-related liver disease; CP, Child-Pugh; HBV, hepatitis B virus; HCV, hepatitis C virus; MASLD, metabolic dysfunction-associated steatotic liver disease.

Age, sex, etiology, CP score, tumor diameter, number of nodules, the presence of distant metastases, and treatment allocation were included in a multivariable Cox regression analysis (Table 3). Allocation of curative-intent treatment was associated with the strongest reduction in mortality risk (hazard ratio [HR] 0.17; 95% CI 0.15-0.19), followed by other tumor-directed treatment (HR 0.37; 95% CI 0.34-0.40). In contrast, the presence of distant metastases (HR 1.99; 95% CI 1.83-2.15) and CP-C (HR 1.90; 95% CI 1.57-2.29) were strongly associated with increased mortality risk. ALD-related HCC was associated with an increased risk of mortality compared to MASLD-related HCC (HR 1.21; 95% CI 1.08-1.35). Similarly, unknown etiology was associated with an increased risk of mortality compared to MASLD-related HCC (HR 1.17; 95% CI 1.05-1.31).

Discussion

Here we present data on incidence, etiology, tumor characteristics, and overall survival of patients with HCC in the Netherlands between 2014 and 2022. The age-adjusted incidence rate rose from 3.5 to 4.1 per 100,000 person-years, corresponding to an AAPC of 2.3%. Although ALD was the most common etiology, MASLD-related HCC showed the strongest increase over time. Incidence rates for other etiologies remained stable throughout the study period.

Our findings build on the timeline of earlier Dutch HCC cohort studies. Between 1989 and 2009, the AAPC in HCC incidence was 0.6%,[19] whereas data from 2009-2016 showed an increase in the age-adjusted incidence rate from 2.9 to 3.7 per 100,000 person-years, corresponding to an AAPC of 3.9%.[3] In the United States, the age-adjusted incidence rate was 5.5 per 100,000 person-years between 2001 and 2021, with an AAPC of 2.5%.[20] In contrast, global incidence declined from 9.0 per 100,000 person-years in 1990 to 6.5 per 100,000 person-years in 2019, largely driven by reductions in East Asia following effective HBV vaccination programs.[4,21]

ALD was the most frequent etiology in our cohort, followed by MASLD. Notably, nearly one-fifth of patients had no recorded underlying risk factor for chronic liver disease, and the etiology was unknown in one-third of the cohort. Globally, HBV and HCV remain the dominant causes of HCC, although non-viral etiologies are increasing.[22,23] The rising incidence of MASLD-related HCC in our cohort aligns with this shift. Nevertheless, some misclassification is likely. Among patients registered without an apparent risk factor for chronic liver disease, 40.7% had at least one cardiometabolic risk factor, suggesting that some may have had undiagnosed MASLD. Due to missing data on obesity, dyslipidemia, and hypertension, the true proportion of MASLD-related HCC may have been underestimated. Similar patterns have been reported previously.[24]

MASLD-related HCC was associated with better overall survival than ALD-related HCC and HCC with unknown etiology. This improved overall survival probability persisted after adjusting for treatment allocation, age, sex, CP score, tumor diameter, number of nodules, and the presence of distant metastases. Overall, curative-intent treatment was the strongest predictor of overall mortality, followed by other tumor-directed treatment, the presence of distant metastases, and CP-C.

The absence of cirrhosis was noted in a substantial proportion of patients. Notably, in the final 3 years of our study period, more than 30% of patients were recorded as not having cirrhosis, consistent with previous studies.[25–27] Patients without cirrhosis presented with larger tumor diameters and more frequently had unifocal disease, in line with earlier findings.[28] Although larger tumors in this group may reflect the absence of surveillance, the proportion receiving curative-intent treatment did not differ between patients with and without cirrhosis in our cohort. The preserved liver function and unifocal nature of tumors in non-cirrhotic patients may partially explain their high eligibility for curative therapy despite later detection.

Table 3. Univariable and multivariable Cox regression analysis of patient and tumor characteristics and mortality (N = 6,526).

		Univariable Cox regression	Multivariable Cox regression
**Variables**	**Patients (N = 6,526)**	**HR (95% CI)**	**HR (95% CI)**
Sex, n (%)			
Men	4,901 (75.1)	Ref.	Ref.
Women	1,625 (24.9)	1.021 (0.958-1.087)	1.000 (0.931-1.075)
Age at diagnosis, years (mean (SD))	69.8 (10.6)	1.029 (1.027-1.032)	1.012 (1.009-1.016)
Etiology, n (%)			
MASLD	711 (10.9)	Ref.	Ref.
ALD	1,322 (20.3)	1.424 (1.280-1.584)	1.208 (1.078-1.352)
HBV	466 (7.1)	1.038 (0.902-1.195)	1.013 (0.869-1.179)
HCV	508 (7.8)	1.007 (0.879-1.154)	0.926 (0.800-1.073)
Other	186 (2.9)	1.138 (0.943-1.374)	0.961 (0.786-1.174)
No risk factor	1,175 (18.0)	1.312 (1.175-1.465)	0.915 (0.800-1.046)
Unknown	2,158 (33.1)	2.109 (1.911-2.329)	1.172 (1.047-1.312)
CP score, n (%)			
No cirrhosis	1,676 (25.7)	Ref.	Ref.
A	1,850 (28.3)	0.732 (0.677-0.791)	0.991 (0.894-1.100)
B	864 (13.2)	1.395 (1.273-1.528)	1.432 (1.269-1.616)
C	250 (3.8)	2.404 (2.086-2.770)	1.895 (1.571-2.286)
Cirrhosis, CP score unknown	259 (4.0)	1.525 (1.316-1.766)	1.336 (1.120-1.595)
Unknown cirrhotic status	1,627 (24.9)	1.777 (1.648-1.917)	1.100 (0.992-1.220)
Tumor diameter, mm (median (IQR))	50 (28, 91)	1.009 (1.008-1.009)	1.005 (1.005-1.006)
Number of nodules, n (%)			
1	2,858 (43.8)	Ref.	Ref.
2	854 (13.1)	1.225 (1.120-1.339)	1.170 (1.067-1.283)
3	390 (6.0)	1.373 (1.219-1.547)	1.146 (1.012-1.297)
Multifocal/ diffuse	1,938 (29.7)	2.930 (2.744-3.129)	1.471 (1.362-1.588)
Unknown	486 (7.4)	2.317 (2.008-2.572)	1.235 (0.969-1.574)
Distant metastasis, n (%)			
No	5,097 (78.1)	Ref.	Ref.
Yes	1,263 (19.4)	3.680 (3.438-3.940)	1.986 (1.831-2.154)
Unknown	166 (2.5)	4.247 (3.630-4.970)	1.511 (1.077-2.120)
Treatment, n (%)			
No tumor-directed	3,045 (46.7)	Ref.	
Curative-intent	1,762 (27.0)	0.093 (0.086-0.101)	0.169 (0.153-0.187)
Other tumor-directed	1,719 (26.3)	0.287 (0.268-0.307)	0.372 (0.344-0.403)

ALD, alcohol-related liver disease; CP, Child-Pugh; HBV, hepatitis B virus; HCC, hepatocellular carcinoma; HCV, hepatitis C virus; MASLD, metabolic dysfunction-associated steatotic liver disease.

This study has several limitations inherent to its retrospective, registry-based design. A considerable proportion of patients had missing data, particularly regarding etiology and cirrhotic status. Patients with an unknown etiology generally presented with larger tumors, less frequently had solitary lesions, were less likely to receive tumor-directed treatment, and had poorer overall survival. This group likely represents patients presenting with more advanced disease, potentially resulting in less extensive diagnostic evaluation, especially in the context of a high comorbidity burden and limited treatment options. Consequently, survival estimates among patients with a documented etiology may be overly optimistic. Given the extent of missing data, comparisons between etiological subgroups should be interpreted with caution and primarily viewed as descriptive. In addition, the data captured by the NCR lack key tumor-specific or clinical variables, including alpha-fetoprotein levels, performance status, detailed comorbidity indices, and quantitatively assessed alcohol consumption. The absence of alcohol consumption data limits insight into potential overlap between MASLD and ALD, which may have led to misclassification. Furthermore, the recent nomenclature change from NAFLD to MASLD in 2023, accompanied by modified diagnostic criteria, may have introduced classification inconsistencies over time.[5] Although registry data are systematically collected, some degree of misclassification cannot be excluded.

The survival analyses should be interpreted as strictly descriptive. Although multivariable Cox regression was performed to adjust for measured covariates, residual confounding is likely. Treatment allocation is strongly influenced by baseline disease severity, liver function, age, and comorbidity, factors that are incompletely captured in the registry. Therefore, the observed associations between treatment allocation and survival should not be interpreted as causal effects. Moreover, the analyses are susceptible to immortal time bias, potentially leading to an overestimation of survival differences between treatment groups. Given the multiple subgroup comparisons performed, the analyses were not designed for formal hypothesis testing. No correction for multiplicity was applied; therefore, the findings should be considered as descriptive patterns rather than confirmatory evidence. A key strength of this study is the use of a large, nationwide, unselected cohort, enabling detailed description of national temporal trends and clinical patterns of HCC.

In conclusion, this nationwide study of HCC in the Netherlands highlights a steadily increasing incidence rate over the past decade, with the most pronounced rise observed in MASLD-related HCC and in patients without a documented underlying risk factor for chronic liver disease. Overall survival remains poor, particularly among patients with an unknown etiology or cirrhotic status, who are likely not recognized as

having underlying liver disease prior to HCC diagnosis and are therefore not enrolled in regular surveillance programs. These findings underscore the need for greater clinical awareness and earlier identification of underlying liver disease to facilitate timely detection, appropriate management, and ultimately improved long-term patient survival.

Affiliations

[1]Department of Vascular Medicine, Amsterdam UMC, University of Amsterdam, Meibergdreef 9, 1105 AZ, Amsterdam, The Netherlands; [2]Department of Gastroenterology and Hepatology, Amsterdam UMC, University of Amsterdam, De Boelelaan 1117, 1081 HV Amsterdam, The Netherlands; [3]Amsterdam Gastroenterology Endocrinology Metabolism (AGEM) Institute, Amsterdam UMC, University of Amsterdam, Meibergdreef 9, 1105 AZ, Amsterdam, The Netherlands; [4]Div. Medical Oncology, Department of Internal Medicine, GROW – Research Institute for Oncology & Reproduction, Maastricht University Medical Center, P. Debyelaan 25, 6229 HX, Maastricht, The Netherlands; [5]Department of Medical Oncology, UMC Groningen, Hanzeplein 1, 9713 GZ Groningen, The Netherlands; [6]Department of Gastroenterology and Hepatology, Radboud University Medical Center, Geert Grooteplein Zuid 10, 6525 GA, Nijmegen, The Netherlands; [7]Department of Gastroenterology and Hepatology, Utrecht University Medical Center, Utrecht, Heidelberglaan 100, 3584 CV, Utrecht, The Netherlands; [8]Department of Medical Oncology, Utrecht University Medical Center, Utrecht University, Utrecht, Heidelberglaan 100, 3584 CV, Utrecht, The Netherlands; [9]Department of Gastroenterology and Hepatology, Erasmus University Medical Center, Dr. Molewaterplein 40, 3015 GD, Rotterdam, The Netherlands; [10]Department of Gastroenterology and Hepatology, Leiden University Medical Center, Albinusdreef 2, 2333 ZA, Leiden, The Netherlands; [11]Department of Gastroenterology, Division of Hepatology, University Medical Center Groningen, Hanzeplein 1, 9713 GZ, Groningen, The Netherlands; [12]Department of Radiology, Amsterdam UMC, University of Amsterdam, De Boelelaan 1117, 1081 HV Amsterdam, The Netherlands; [13]Department of Research and Development, Netherlands Comprehensive Cancer Organisation (IKNL), Rijnkade 5, 3511 LC, Utrecht, the Netherlands

Abbreviations

AAPC, average annual percentage change; ALD, alcohol-related liver disease; CVD, cardiovascular disease; CP, Child–Pugh; HBV, hepatitis B virus; HCC, hepatocellular carcinoma; HCV, hepatitis C virus; HR, hazard ratio; MASLD, metabolic dysfunction-associated liver disease; NCR, Netherlands Cancer Registry.

Financial support

This study was financially supported by the MDLS NLA2 grant (WO-21-06) and the MLDS NAFLD-NL grant (WOO 20-07). The funder had no role in the design, conduct, or reporting of the study.

Conflicts of interest

No conflict of interest: KvS, KvE, JdV-G, HK, ET, JB, NHM, DS, MC, FvV, OD, JD, MT, AH, LvdG, RT.

Please refer to the accompanying ICMJE disclosure forms for further details.

Authors' contributions

Conceptualization: KvS, KvE, JdV-G, HK, ET, JB, NHM, DS, MC, FvV, OD, JD, MT, AH, LvdG, RT. Data curation: KvS, LvdG. Formal analysis: KvS, LvdG. Funding acquisition: JdV-G, HK, ET, JB, NHM, DS, MC, FvV, OD, JD, MT, AH, LvdG, RT. Investigation: KvS, KvE, LvdG, RT. Methodology: KvS, KvE, LvdG, RT. Project administration: KvS, RT. Software: not applicable. Resources: LvdG. Supervision: JdV-G, HK, ET, JB, NHM, DS, MC, FvV, OD, JD, MT, AH, LvdG, RT. Validation: not applicable. Visualization: K*vs.* Writing – original draft: KvS, KvE, LvdG, RT. Writing – review & editing: KvS, KvE, JdV-G, HK, ET, JB, NHM, DS, MC, FvV, OD, JD, MT, AH, LvdG, RT.

Data availability

Data that support the findings of this study originate from the Netherlands Cancer Registry (NCR), maintained by the Netherlands Comprehensive Cancer Organisation (IKNL). These data are not publicly available due to privacy and confidentiality restrictions. Access can be requested via IKNL's Data Request procedure (https://iknl.nl/en/ncr/apply-for-data).

Acknowledgements

The authors thank the registration team of the Netherlands Comprehensive Cancer Organisation (IKNL) for the collection of data for the Netherlands Cancer Registry.

Supplementary data

Supplementary data to this article can be found online at https://doi.org/10.1016/j.jhepr.2026.101924.

References

Author names in bold designate shared co-first authorship

[1] Sung H, Ferlay J, Siegel RL, et al. Global cancer statistics 2020: GLOBOCAN estimates of incidence and mortality worldwide for 36 cancers in 185 countries. CA Cancer J Clin 2021;71:209–249. https://doi.org/10.3322/caac.21660.

[2] Llovet JM, Zucman-Rossi J, Pikarsky E, et al. Hepatocellular carcinoma. Nat Rev Dis Primers 2016;2. https://doi.org/10.1038/nrdp.2016.18.

[3] Reinders MTM, van Meer S, Burgmans MC, et al. Trends in incidence, diagnosis, treatment and survival of hepatocellular carcinoma in a low-incidence country: data from The Netherlands in the period 2009–2016. Eur J Cancer 2020;137:214–223. https://doi.org/10.1016/j.ejca.2020.07.008.

[4] Yang J, Pan G, Guan L, et al. The burden of primary liver cancer caused by specific etiologies from 1990 to 2019 at the global, regional, and national levels. Cancer Med 2022;11:1357–1370. https://doi.org/10.1002/cam4.4530.

[5] Rinella ME, Lazarus JV, Ratziu V, et al. A multisociety Delphi consensus statement on new fatty liver disease nomenclature. Hepatology 2023;78:1966–1986. https://doi.org/10.1097/HEP.0000000000000520.

[6] Paik JM, Golabi P, Younossi Y, et al. Changes in the global burden of chronic liver diseases from 2012 to 2017: the growing impact of NAFLD. Hepatology 2020;72:1605–1616. https://doi.org/10.1002/hep.31173.

[7] Younossi ZM, Koenig AB, Abdelatif D, et al. Global epidemiology of nonalcoholic fatty liver disease—meta-analytic assessment of prevalence, incidence, and outcomes. Hepatology 2016;64:73–84. https://doi.org/10.1002/hep.28431.

[8] Diehl AM, Day C. Cause, pathogenesis, and treatment of nonalcoholic steatohepatitis. New Engl J Med 2017;377:2063–2072. https://doi.org/10.1056/nejmra1503519.

[9] Estes C, Anstee QM, Arias-Loste MT, et al. Modeling NAFLD disease burden in China, France, Germany, Italy, Japan, Spain, United Kingdom, and United States for the period 2016–2030. J Hepatol 2018;69:896–904. https://doi.org/10.1016/j.jhep.2018.05.036.

[10] Younossi ZM, Otgonsuren M, Henry L, et al. Association of nonalcoholic fatty liver disease (NAFLD) with hepatocellular carcinoma (HCC) in the United States from 2004 to 2009. Hepatology 2015;62:1723–1730. https://doi.org/10.1002/hep.28123.

[11] Kim NG, Nguyen PP, Dang H, et al. Temporal trends in disease presentation and survival of patients with hepatocellular carcinoma: a real-world experience from 1998 to 2015. Cancer 2018;124:2588–2598. https://doi.org/10.1002/cncr.31373.

[12] Hester D, Golabi P, Paik J, et al. Among medicare patients with hepatocellular carcinoma, non-alcoholic fatty liver disease is the most common etiology and cause of mortality. J Clin Gastroenterol 2020;54:459–467. https://doi.org/10.1097/MCG.0000000000001172.

[13] Mittal S, Sada YH, El-Serag HB, et al. Temporal trends of nonalcoholic fatty liver disease-related hepatocellular carcinoma in the veteran affairs population. Clin Gastroenterol Hepatol 2015;13:594–601.e1. https://doi.org/10.1016/j.cgh.2014.08.013.

[14] Tan DJH, Ng CH, Lin SY, et al. Clinical characteristics, surveillance, treatment allocation, and outcomes of non-alcoholic fatty liver disease-related hepatocellular carcinoma: a systematic review and meta-analysis. Lancet Oncol 2022;23:521–530. https://doi.org/10.1016/S1470-2045(22)00078-X.

[15] Vitellius C, Desjonqueres E, Lequoy M, et al. MASLD-related HCC: multicenter study comparing patients with and without cirrhosis. JHEP Rep 2024;6:101160. https://doi.org/10.1016/j.jhepr.2024.101160.
[16] World Health Organisation. International classification of diseases for Oncology (ICD-O). 3rd ed 2013. 1st revision.
[17] von Elm E, Altman DG, Egger M, et al. The Strengthening the Reporting of Observational Studies in Epidemiology (STROBE) statement: guidelines for reporting observational studies. Lancet 2007;370:1453–1457. https://doi.org/10.1016/S0140-6736(07)61602-X.
[18] Eurostat. Revision of the European standard population - report of eurostat's task force - 2013 edition. 2013. Eurostat.
[19] Witjes CDM, Karim-Kos HE, Visser O, et al. Hepatocellular carcinoma in a low-endemic area: rising incidence and improved survival. Eur J Gastroenterol Hepatol 2012;24:450–457. https://doi.org/10.1097/MEG.0b013e32835030ce.
[20] Sokale IO, Rosales O, Thrift AP, et al. Differences in hepatocellular carcinoma incidence trends across US census divisions, 2001 to 2021. Cancers (Basel) 2025;17. https://doi.org/10.3390/cancers17091431.
[21] Liu Z, Mao X, Jiang Y, et al. Changing trends in the disease burden of primary liver cancer caused by specific etiologies in China. Cancer Med 2019;8:5787–5799. https://doi.org/10.1002/cam4.2477.
[22] **Liu Y**, **Zheng J**, Hao J, et al. Global burden of primary liver cancer by five etiologies and global prediction by 2035 based on global burden of disease study 2019. Cancer Med 2022;11:1310–1323. https://doi.org/10.1002/cam4.4551.
[23] Singal AG, Kanwal F, Llovet JM. Global trends in hepatocellular carcinoma epidemiology: implications for screening, prevention and therapy. Nat Rev Clin Oncol 2023;20:864–884. https://doi.org/10.1038/s41571-023-00825-3.
[24] Dyson J, Jaques B, Chattopadyhay D, et al. Hepatocellular cancer: the impact of obesity, type 2 diabetes and a multidisciplinary team. J Hepatol 2014;60:110–117. https://doi.org/10.1016/j.jhep.2013.08.011.
[25] Cranford HM, Jones PD, Wong RJ, et al. Hepatocellular carcinoma etiology drives survival outcomes: a population-based analysis. Cancer Epidemiol Biomarkers Prev 2024. https://doi.org/10.1158/1055-9965.epi-24-0626.
[26] Engstrand J, Stål P, Gilg S, et al. Hepatocellular carcinoma in cirrhotic versus non-cirrhotic liver: treatment and survival differences in a nationwide cohort. Scand J Surg 2024;113:120–130. https://doi.org/10.1177/14574969231220179.
[27] Armandi A, Rosso C, Caviglia GP, et al. An updated overview on hepatocellular carcinoma in patients with Metabolic dysfunction-Associated Steatotic Liver Disease: trends, pathophysiology and risk-based surveillance. Metabolism 2025;162. https://doi.org/10.1016/j.metabol.2024.156080.
[28] Tan DJH, Ng CH, Lin SY, et al. Clinical characteristics, surveillance, treatment allocation, and outcomes of non-alcoholic fatty liver disease-related hepatocellular carcinoma: a systematic review and meta-analysis. Lancet Oncol 2022;23:521–530. https://doi.org/10.1016/S1470-2045(22)00078-X.

Keywords: Hepatocellular carcinoma; temporal trends; epidemiology; nationwide; registry.
Received 16 June 2025; received in revised form 1 June 2026; accepted 2 June 2026; Available online 23 June 2026

Journal of Hepatology, Volume *8*

Supplemental information

Increasing incidence and changing trends in etiology & survival rates in HCC in a European cohort

Koen C. van Son, Kirsi M.A. van Eekhout, Judith de Vos-Geelen, Heinz-Josef Klümpen, Eric T.T.L. Tjwa, Joep de Bruijne, Nadia Haj Mohammad, Dave Sprengers, Minneke J. Coenraad, Frederike G.I. van Vilsteren, Otto M. van Delden, Joost P.H. Drenth, Maarten E. Tushuizen, Adriaan G. Holleboom, Lydia G. van der Geest, R. Bart Takkenberg, and for the Dutch Hepatocellular & Cholangio Carcinoma Group (DHCG)

Increasing incidence and changing trends in etiology & survival rates in HCC in a European cohort

Koen C. van Son, Kirsi M.A. van Eekhout, Judith de Vos-Geelen, Heinz-Josef Klümpen, Eric T.T.L. Tjwa, Joep de Bruijne, Nadia Haj Mohammad, Dave Sprengers, Minneke J. Coenraad, Frederike G.I. van Vilsteren, Otto M. van Delden, Joost P.H. Drenth, Maarten E. Tushuizen, Adriaan G. Holleboom, Lydia G. van der Geest, R. Bart Takkenberg for the Dutch Hepatocellular & Cholangio Carcinoma Group (DHCG)

Table S1..2

Table S2..4

Table S3..5

Table S4..7

Table S5..9

Supplementary tables

Included morphology codes			
8000	Neoplasm, not otherwise specified (no microscopic verification)		
8001	Malignant tumor cells		
8170	HCC not further specified		
8171	Fibrolamellar HCC		
8172	Scirrhous HCC		
8173	Sarcomatoid HCC		
8174	Clear cell HCC		
8175	Pleomorphic HCC		
Included NCR variables			
Variable	NCR variable	Study variable	
Year of diagnosis	Year	Year	
Sex	Men	Men	
	Women	Women	
T2DM	Yes	Yes	
	No	No	
	Unknown or no available data	Unknown	
CVD	Myocardial infarction/CVD/congestive heart disease or peripheral heart disease present	Yes	
	Myocardial infarction/CVD/congestive heart disease and peripheral heart disease absent	No	
	Myocardial infarction/CVD/congestive heart disease and peripheral heart disease unknown or no available data	Unknown	
Age at diagnosis	Year	Year	
Etiology			Hierarchical order
	Hepatitis B	HBV	1
	Hepatitis C	HCV	2
	Alcoholic cirrhosis	ALD	3
	NAFLD/NASH	MASLD	4
	Auto-immune hepatitis/primary biliary cirrhosis	Other	5
	Hemochromatosis/M. Wilson/porphyria	Other	6
	No risk factor	No risk factor	7
	Liver cirrhosis with unknown etiology/unknown	Unknown	NA

	cause/cryptogenic cirrhosis or no available data	
Cirrhosis	Liver cirrhosis, no CP score	Yes
	CP-A, CP-B, or CP-C	Yes
	CP-0	No
	Unknown or no available data	Unknown
CP score	0	No cirrhosis
	Liver cirrhosis, no CP score	Cirrhosis, CP unknown
	Unknown or no available data	Unknown
	A	A
	B	B
	C	C
Tumor diameter	mm	mm
Number of nodules	1	1
	2	2
	3	3
	Multifocal/diffuse	Multifocal/diffuse
	No available data	Unknown
Distant metastasis	Yes	Yes
	No	No
	Unknown	Unknown
Treatment	Resection, primary tumor destruction using RFA or MWA, or liver transplantation	Curative-intent
	Tumor-directed tumor other than resection, primary tumor destruction using RFA or MWA, or liver transplantation	Other tumor-directed
	No tumor-directed treatment	No tumor-directed

Table S1: Overview of the included data. HCC, hepatocellular carcinoma; NCR, Netherlands Cancer Registry; T2DM, type 2 diabetes mellitus; CVD, cardiovascular disease; NAFLD/NASH, non-alcoholic fatty liver disease/non-alcoholic steatohepatitis; MASLD, metabolic dysfunction-associated steatotic liver disease; ALD, alcoholic liver disease; HBV, hepatitis B virus; HCV, hepatitis C virus; CP, Child-Pugh; RFA, radiofrequency ablation; MWA, microwave ablation.

	Age-adjusted incidence rate (per 100.000 person-years)									AAPC (95% CI)
	2014	2015	2016	2017	2018	2019	2020	2021	2022	
HCC	3.53	3.93	3.70	4.16	4.40	4.37	4.27	4.61	4.12	2.3 (0.9-3.7)
Stratified by etiology										
MASLD	0.29	0.25	0.29	0.37	0.61	0.48	0.60	0.60	0.48	8.3 (2.9-13.7)
ALD	0.78	0.75	0.62	0.78	0.87	0.95	0.90	0.92	0.83	2.1 (-1.3-5.5)
HBV	0.37	0.35	0.21	0.33	0.36	0.33	0.21	0.24	0.25	-4.5 (-11.4-2.4)
HCV	0.31	0.41	0.27	0.32	0.37	0.32	0.28	0.29	0.27	-2.5 (-6.7-1.6)
Other	0.11	0.12	0.07	0.12	0.10	0.13	0.17	0.12	0.11	1.9 (-5.6-9.4)
No risk factor	0.55	0.80	0.53	0.65	0.62	0.71	0.87	0.97	0.99	6.7 (2.4-10.9)
Unknown	1.12	1.24	1.70	1.58	1.47	1.45	1.24	1.47	1.19	1.7 (-0.6-4.1)

Table S2: Age-adjusted incidence rate and average annual percentage change of HCC in the Netherlands between 2014 and 2022 stratified by etiology (n=6,526). AAPC = average annual percent change; HCC = hepatocellular carcinoma; MASLD = metabolic dysfunction-associated steatotic liver disease; ALD = alcohol-related liver disease, HBV = hepatitis B virus, HCV = hepatitis C virus.

		Etiology							
Variable		MASLD	ALD	HBV	HCV	Other	No risk factor	Unknown	p-value
n		711	1,322	466	508	186	1,175	2,158	
Year of diagnosis, n (%)	2014	46 (6.5)	128 (9.7)	63 (13.5)	53 (10.4)	18 (9.7)	87 (7.4)	175 (8.1)	<0.001*
	2015	41 (5.8)	125 (9.5)	60 (12.9)	70 (13.8)	21 (11.3)	129 (11.0)	200 (9.3)	
	2016	50 (7.0)	107 (8.1)	36 (7.7)	48 (9.4)	12 (6.5)	88 (7.5)	283 (13.1)	
	2017	64 (9.0)	137 (10.4)	58 (12.4)	57 (11.2)	20 (10.8)	110 (9.4)	268 (12.4)	
	2018	107 (15.0)	154 (11.6)	63 (13.5)	66 (13.0)	18 (9.7)	108 (9.2)	251 (11.6)	
	2019	88 (12.4)	174 (13.2)	58 (12.4)	58 (11.4)	23 (12.4)	126 (10.7)	259 (12.0)	
	2020	110 (15.5)	165 (12.5)	38 (8.2)	51 (10.0)	31 (16.7)	158 (13.4)	224 (10.4)	
	2021	113 (15.9)	174 (13.2)	44 (9.4)	54 (10.6)	22 (11.8)	179 (15.2)	272 (12.6)	
	2022	92 (12.9)	158 (12.0)	46 (9.9)	51 (10.0)	21 (11.3)	190 (16.2)	226 (10.5)	
Sex, n men (%)		504 (70.9)	1,120 (84.7)	401 (86.1)	387 (76.2)	120 (64.5)	856 (72.9)	1,513 (70.1)	<0.001*
T2DM, n (%)	Yes	353 (49.6)	335 (25.3)	78 (16.7)	74 (14.6)	40 (21.5)	361 (30.7)	553 (25.6)	<0.001*
	No	154 (21.7)	541 (40.9)	191 (41.0)	237 (46.7)	81 (43.5)	471 (40.1)	705 (32.7)	
	Unknown	204 (28.7)	446 (33.7)	197 (42.3)	197 (38.8)	65 (34.9)	343 (29.2)	900 (41.7)	
CVD, n (%)	Yes	132 (18.6)	181 (13.7)	41 (8.8)	46 (9.1)	26 (14.0)	254 (21.6)	373 (17.3)	<0.001*
	No	375 (52.7)	695 (52.6)	228 (48.9)	265 (52.2)	95 (51.1)	578 (49.2)	885 (41.0)	
	Unknown	204 (28.7)	446 (33.7)	197 (42.3)	197 (38.8)	65 (34.9)	343 (29.2)	900 (41.7)	
Age at diagnosis, years (mean (SD))		70.0 (8.2)	68.4 (7.7)	61.1 (12.4)	63.0 (8.2)	67.2 (12.2)	71.9 (11.4)	73.0 (10.4)	<0.001**
Cirrhosis, n (%)	Yes	538 (75.7)	1,106 (83.7)	274 (58.8)	387 (76.2)	118 (63.4)	83 (7.1)	717 (33.2)	<0.001*
	No	98 (13.8)	37 (2.8)	110 (23.6)	54 (10.6)	37 (19.9)	971 (82.6)	369 (17.1)	
	Unknown	75 (10.5)	179 (13.5)	82 (17.6)	67 (13.2)	31 (16.7)	121 (10.3)	1,072 (49.7)	
Child-Pugh score, n (%)	A	359 (50.5)	563 (42.6)	182 (39.1)	263 (51.8)	73 (39.2)	52 (4.4)	358 (16.6)	<0.001*
	B	135 (19.0)	345 (26.1)	58 (12.4)	78 (15.4)	33 (17.7)	16 (1.4)	199 (9.2)	
	C	23 (3.2)	119 (9.0)	18 (3.9)	29 (5.7)	6 (3.2)	2 (0.2)	53 (2.5)	
	Cirrhosis, CP score unknown	21 (3.0)	79 (6.0)	16 (3.4)	17 (3.3)	6 (3.2)	13 (1.1)	107 (5.0)	
	No cirrhosis	98 (13.8)	37 (2.8)	110 (23.6)	54 (10.6)	27 (19.9(	971 (82.6)	369 (17.1)	
	Unknown cirrhotic status	75 (10.5)	179 (13.5)	82 (17.6)	67 (13.2)	31 (16.7)	121 (10.3)	1,072 (49.7)	

Tumor diameter, mm (median (IQR))		34 (23, 58)	36 (23, 60)	45 (25, 88)	34 (22, 60)	40 (27, 76)	85 (50, 130)	67 (38, 102)	<0.001***
Number of nodules, n (%)	1	359 (50.5)	539 (40.8)	235 (50.4)	237 (46.7)	98 (52.7)	623 (53.0)	767 (35.5)	<0.001*
	2	123 (17.3)	225 (17.0)	69 (14.8)	67 (13.2)	33 (17.7)	128 (10.9)	209 (9.7)	
	3	53 (7.5)	94 (7.1)	25 (5.4)	47 (9.3)	14 (7.5)	54 (4.6)	103 (4.8)	
	Multifocal/ diffuse	159 (22.4)	418 (31.6)	130 (27.9)	145 (28.5)	36 (19.4)	342 (29.1)	708 (32.8)	
	Unknown	17 (2.4)	46 (3.5)	7 (1.5)	12 (2.4)	5 (2.7)	28 (2.4)	371 (17.2)	
Distant metastasis, n (%)	Yes	63 (8.9)	171 (12.9)	93 (20.0)	58 (11.4)	22 (11.8)	281 (23.9)	575 (26.6)	<0.001*
		643 (90.4)	1,131 (85.6)	367 (78.8)	445 (87.6)	162 (87.1)	875 (74.5)	1,474 (68.3)	
	Unknown	5 (0.7)	20 (1.5)	6 (1.3)	5 (1.0)	2 (1.1)	19 (1.6)	109 (5.1)	
Treatment, n (%)	Curative-intent	278 (39.1)	384 (29.0)	174 (37.3)	196 (38.6)	66 (35.5)	331 (28.2)	333 (15.4)	<0.001*
	Other tumor-directed	244 (34.3)	383 (29.0)	141 (30.3)	129 (25.4)	50 (26.9)	351 (29.9)	421 (19.5)	
	No tumor-directed	189 (26.6)	555 (42.0)	151 (32.4)	183 (36.0)	70 (37.6)	493 (42.0)	1,404 (65.1)	

Table S3: Patient characteristics stratified by etiology (n=6,526). SD = standard deviation; IQR = interquartile range; T2DM = type 2 diabetes mellitus; CVD = cardiovascular disease; MASLD = metabolic-dysfunction associated steatotic liver disease, ALD = alcohol-related liver disease, HBV = hepatitis B virus; HCV = hepatitis C virus; CP, Child-Pugh. * Chi-square test; ** One-way ANOVA; *** Kruskal-Wallis test.

		Presence of cirrhosis				
Variable		Yes	No	p-value (Yes vs. No)	Unknown	p-value (Yes vs. No vs. Unknown)
n		3,223	1,676		1,627	
Year of diagnosis, n (%)	2014	246 (7.6)	103 (6.1)	<0.001*	221 (13.6)	<0.001*
	2015	267 (8.3)	165 (9.8)		214 (13.2)	
	2016	263 (8.2)	101 (6.0)		240 (14.8)	
	2017	322 (10.0)	152 (9.1)		240 (14.8)	
	2018	371 (11.5)	165 (9.8)		231 (14.2)	
	2019	418 (13.0)	179 (10.7)		189 (11.6)	
	2020	433 (13.4)	244 (14.6)		100 (6.1)	
	2021	454 (14.1)	298 (17.8)		106 (6.5)	
	2022	449 (13.9)	269 (16.1)		66 (4.1)	
Sex, n men (%)		2,522 (78.3)	1,223 (73.0)	<0.001*	1,156 (71.1)	<0.001*
T2DM, n (%)	Yes	1,011 (31.4)	489 (29.2)	<0.001*	294 (18.1)	0.006*
	No	1,252 (38.8)	730 (43.6)		398 (24.5)	
	Unknown	960 (29.8)	457 (27.3)		935 (57.5)	
CVD, n (%)	Yes	500 (15.5)	359 (21.4)	<0.001*	194 (11.9)	<0.001*
	No	1,763 (54.7)	860 (51.3)		498 (30.6)	
	Unknown	960 (29.8)	457 (27.3)		934 (57.5)	
Age at diagnosis, years (mean (SD))		1,763 (54.7)	860 (51.3)	<0.001**	498 (30.6)	<0.001**
Etiology, n (%)	MASLD	538 (16.7)	98 (5.8)	<0.001*	75 (4.6)	<0.001*
	ALD	1,106 (16.7)	37 (2.2)		179 (11.0)	
	HBV	274 (8.5)	110 (6.6)		82 (5.0)	
	HCV	387 (12.0)	54 (3.2)		67 (4.1)	
	Other	118 (3.7)	37 (2.2)		31 (1.9)	
	No risk factor	83 (2.6)	971 (57.9)		121 (7.4)	
	Unknown	717 (22.2)	369 (22.0)		1,072 (65.9)	
Tumor diameter, mm (median (IQR))		36 (23, 62)	80 (46, 122)	<0.001***	70 (36, 100)	<0.001***
Number of nodules, n (%)	1	1,391 (43.2)	923 (55.1)	<0.001*	544 (33.4)	<0.001*
	2	528 (16.4)	171 (10.2)		155 (9.5)	

	3	255 (7.9)	81 (4.8)		54 (3.3)	
	Multifocal/ diffuse	966 (30.0)	467 (27.9)		505 (31.0)	
	Unknown	83 (2.6)	34 (2.0)		369 (22.7)	
Distant metastasis, n (%)	Yes	431 (13.4)	379 (22.6)	<0.001*	453 (27.8)	<0.001*
	No	2,757 (85.5)	1,266 (75.5)		1,074 (66.0)	
	Unknown	35 (1.1)	31 (1.8)		100 (6.1)	
Treatment, n (%)	Curative-intent	1,002 (31.1)	505 (30.1)	p=0.004*	255 (15.7)	<0.001*
	Other tumor-directed	1,030 (32.0)	475 (28.3)		214 (13.2)	
	No tumor-directed	1,191 (37.0)	696 (41.5)		1,158 (71.2)	

Table S4: Patient characteristics stratified by cirrhotic status (n=6,526). SD = standard deviation; IQR = interquartile range; T2DM = type 2 diabetes mellitus; CVD = cardiovascular disease; MASLD = metabolic-dysfunction associated steatotic liver disease, ALD = alcohol-related liver disease, HBV = hepatitis B virus; HCV = hepatitis C virus. * Chi-square test; ** Unpaired t-test; *** Mann-Whitney U-test.

	1-year (%)	3-year (%)	5-year (%)	Log-rank test
Overall	46.5	26.1	18.8	-
Etiology				
MASLD	65.3	37.9	24.3	Ref.
ALD	50.0	24.7	16.1	<0.001
HBV	54.6	37.5	30.6	0.626
HCV	62.0	39.7	31.1	0.784
Other	56.8	33.1	25.6	0.194
No risk factor	48.8	28.9	21.9	<0.001
Unknown	30.7	15.5	10.6	<0.001
Child-Pugh score				
No cirrhosis	49.7	30.6	23.8	Ref.
A	69.8	40.5	27.0	<0.001
B	37.4	17.3	12.4	<0.001
C	17.9	9.7	8.6	<0.001
Cirrhosis, CP score unknown	35.5	18.7	14.6	<0.001
Unknown cirrhotic status	27.8	13.9	10.2	<0.001

Table S5: 1-, 3- and 5-year overall survival rate for the entire study population (n=6,526), and stratified for etiology (n=6,526) and Child-Pugh score (n=6,526) using long-rank test. MASLD = metabolic-dysfunction associated steatotic liver disease, ALD = alcohol-related liver disease, HBV = hepatitis B virus; HCV = hepatitis C virus.
